# A 3-D virtual human thermoregulatory model to predict whole-body and organ-specific heat-stress responses

**DOI:** 10.1007/s00421-021-04698-1

**Published:** 2021-06-05

**Authors:** Ginu Unnikrishnan, Rajeev Hatwar, Samantha Hornby, Srinivas Laxminarayan, Tushar Gulati, Luke N. Belval, Gabrielle E. W. Giersch, Josh B. Kazman, Douglas J. Casa, Jaques Reifman

**Affiliations:** 1 Department of Defense Biotechnology High Performance Computing Software Applications Institute, Telemedicine and Advanced Technology Research Center, United States Army Medical Research and Development Command, FCMR-TT, 504 Scott Street, Fort Detrick, MD 21702-5012 USA; 2 The Henry M. Jackson Foundation for the Advancement of Military Medicine, Inc., 6720A Rockledge Drive, Bethesda, MD 20817 USA; 3 Korey Stringer Institute, University of Connecticut, 2095 Hillside Road U-1110, Storrs, CT 06269 USA; 4 Consortium for Health and Military Performance, Department of Military and Emergency Medicine, F. Edward Hébert School of Medicine, Uniformed Services University, Bethesda, MD 20814 USA

**Keywords:** Core body temperature, Environmental heat stress, Exertional heat stress, Finite element model, Heat illness

## Abstract

**Objective:**

This study aimed at assessing the risks associated with human exposure to heat-stress conditions by predicting organ- and tissue-level heat-stress responses under different exertional activities, environmental conditions, and clothing.

**Methods:**

In this study, we developed an anatomically detailed three-dimensional thermoregulatory finite element model of a 50th percentile U.S. male, to predict the spatiotemporal temperature distribution throughout the body. The model accounts for the major heat transfer and thermoregulatory mechanisms, and circadian-rhythm effects. We validated our model by comparing its temperature predictions of various organs (brain, liver, stomach, bladder, and esophagus), and muscles (vastus medialis and triceps brachii) under normal resting conditions (errors between 0.0 and 0.5 °C), and of rectum under different heat-stress conditions (errors between 0.1 and 0.3 °C), with experimental measurements from multiple studies.

**Results:**

Our simulations showed that the rise in the rectal temperature was primarily driven by the activity level (~ 94%) and, to a much lesser extent, environmental conditions or clothing considered in our study. The peak temperature in the heart, liver, and kidney were consistently higher than in the rectum (by ~ 0.6 °C), and the entire heart and liver recorded higher temperatures than in the rectum, indicating that these organs may be more susceptible to heat injury.

**Conclusion:**

Our model can help assess the impact of exertional and environmental heat stressors at the organ level and, in the future, evaluate the efficacy of different whole-body or localized cooling strategies in preserving organ integrity.

**Supplementary Information:**

The online version contains supplementary material available at 10.1007/s00421-021-04698-1.

## Introduction

Heat-related illnesses induced by strenuous physical activity, exposure to adverse environmental conditions, or their combination necessarily involve an increase in core body temperature (Epstein and Roberts 2011; Bouchama and Knochel 2002; Epstein et al. 2012). Heat illness can range from less severe muscle cramps to heat exhaustion and to potentially life-threatening heat stroke (Bouchama and Knochel 2002; Epstein et al. 2012; Varghese et al. 2005), where heat-induced cytotoxicity initiates a systemic inflammatory response that may result in multi-organ failure and death (Varghese et al. 2005). While measurements of core body temperature through a rectal probe can help assess the severity of the body’s heat-stress state in total, they may underestimate localized heat loads and the resulting peak temperatures in vital organs, such as the liver and the brain (Jardine 2007; Wang et al. 2014; Cheshire 2016), masking the actual risk of organ injury. Because it is not feasible to measure organ temperature in humans while performing strenuous physical activity, one way to address this challenge is to use computational models to characterize the spatiotemporal distribution of temperatures throughout the entire body resulting from exertional and environmental heat stressors and use this information to infer potential organ-specific injury.

Numerous computational models of human thermoregulation have been developed over the past 50 years. However, the majority of these models are not anatomically realistic (Gagge 1973; Gagge 1971; Nishi and Gagge 1971; Stolwijk 1971; Fiala et al. 1998, 1999, 2001, 2012) as they represent the entire human body either as a single segment (Gagge 1973, 1971; Nishi and Gagge 1971) or as being composed of multiple segments (Stolwijk 1971; Fiala et al. 1998, 1999, 2001, 2012). For example, Gagge and coworkers modeled the entire human body as a single segment with two concentric cylinders, one to represent the body core and the other the outer layer of the skin (Gagge 1973, 1971; Nishi and Gagge 1971). In contrast, the multiple-segment models represent each part of the body, such as the arms and the legs, as a separate segment, each consisting of one or more concentric cylinders (Stolwijk 1971; Fiala et al. 1998, 1999, 2001, 2012). While the single- and multiple-segment models have been shown to reasonably predict core body temperature as well as skin temperature under different heat-stress conditions (Fiala et al. 1998, 2001), they inherently lack the spatial resolution to predict temperature responses at the organ and tissue levels (Nelson et al. 2009), which limits their applicability.

To overcome these limitations, Nelson et al. and Bernardi et al. separately developed voxel-based, anatomically detailed thermoregulatory models to predict the thermal response in humans exposed to high environmental temperatures (Nelson et al. 2009) and radio frequency radiation (Bernardi et al. 2003), respectively. In this formulation, they first divided the entire human body into equal-sized volume elements, or voxels, and then, using a finite difference-time domain algorithm, applied macroscopic energy balance equations to each voxel to compute the spatiotemporal distribution of temperatures throughout the body. While this voxel-based approach allows for temperature predictions at each internal organ, including the skin, it is unclear the extent to which the relatively large length of the voxel elements (2 mm for (Nelson et al. 2009), and 5 or 6 mm for (Bernardi et al. 2003)) affects the accuracy of the temperature predictions. In addition, neither model has been used to quantify the effects of exertional heat stress, which is known to be the primary driver for increases in core body temperature when compared to environmental stressors (Stolwijk 1971; Sawka et al. 1993; Nielsen and Nielsen 1962).

Previously, we developed a three-dimensional (3-D), anatomically detailed thermoregulatory finite element (FE) model of a rat, where we demonstrated the ability to accurately predict the spatiotemporal temperature distributions throughout the animal for a whole host of environmental as well as exertional stress conditions, including cooling (Rakesh et al. 2013, 2014). Here, we extended this framework and developed a FE model for a human—a 3-D thermoregulatory virtual human model—using an anatomically realistic description of a 50th percentile U.S. male that included 25 major organs, such as the brain, heart, lungs, liver, intestines, as well as the skeletal system, with FE sizes as small as 0.04 µm. The model takes environmental conditions, physical activity, and clothing as inputs and predicts the spatiotemporal distribution of temperatures in the entire virtual human as an output. The model accounts for the transfer of energy within the body through macroscopic energy balance equations, heat transfer from the body to the environment through convection, radiation, respiration, and perspiration, the major thermoregulatory mechanisms of the human body, including shivering, sweating, as well as vasoconstriction and vasodilation, and the day-night cycle of temperature changes due to circadian-rhythm effects. We validated our model by comparing our predictions of organ, muscle, and rectal temperatures [which is often used as a surrogate for core body temperature under heat-stress conditions (Casa et al. 2007, 2015)] with experimental measurements. For brain, liver, stomach, bladder, esophagus, and muscles (vastus medalis and triceps brachii), we compared our model predictions against temperature measurements obtained under normal resting conditions. For rectal temperature validation, we used data from three separate studies encompassing a range of time-varying exertional activity levels, different values of atmospheric temperature, relative humidity, and wind speed, as well as different clothing conditions.

## Materials and methods

### Experimental protocol

We used data from two types of studies to validate the 3-D virtual human thermoregulatory model. The first type comprised of experiments conducted at normal resting conditions, where we assessed the temperature of different organs and muscles, whereas the second type comprised of heat-stress challenges, where we assessed collected rectal-temperature measurements. Next, we briefly describe the various studies used for model validation purposes. We refer the readers to the original articles for additional information on the experimental studies (Graf 1959; Ilsley et al. 1983; Taylor et al. 2014; Kenny et al. 2006).

#### Study 1: Organ and muscle temperature measurements during normal resting conditions

##### *Study* 1a: Brain

Eight healthy men [mean age (standard deviation), 26.9 (4.6) years] consented to participate in a study to measure their brain temperature by proton magnetic resonance spectroscopy, using a 3-T MRI scanner (Magnetom Skyra, Siemens Healthcare, Erlangen, Germany) (Onitsuka et al. 2018). For each subject, the temperature was measured in a voxel of interest (VOI) of size 20 × 30 × 20 mm^3^ located in the frontal cortex of the brain, where most of the temperature measurements were recorded between 5:00 and 7:00 p.m. (private communication with Sumire Onitsuka, September 13, 2020).

##### *Study* 1b: Liver and stomach

The study consisted of 75 men who underwent a diagnostic liver biopsy (Graf 1959). The results were either normal or indicated a slight to moderately fatty liver, except for two cases where the patients had incipient cirrhosis. The liver and stomach temperatures were recorded during the morning hours. For liver temperature measurements, copper-constantan thermocouples, with a reported accuracy of ± 0.1 °C, were inserted into the right liver lobe using the Vim-Silverman-Boecker biopsy needle to a depth ranging between 8 and 22 cm. For stomach temperature measurements, a gastric tube containing a copper-constantan thermocouple was passed down into the stomach.

##### *Study* 1c: Bladder

Five subjects participated in this study, in which their bladder temperatures were recorded soon after they were anesthetized for a major vascular surgery (Ilsley et al. 1983). Temperature measurements were carried out using a Foley catheter (Cath-temp Foley Catheter, LaBarge Inc., St. Louis, Missouri, USA), which has a reported accuracy of ± 0.2 °C.

##### *Study* 1d: Esophagus

Esophageal temperatures were obtained from a total of 126 subjects involved in 15 different studies, as reported by Taylor et al*.* (Taylor et al. 2014).

##### *Study* 1e: Muscle

Seven healthy men [mean age (standard deviation), 25 (5) years] participated in a study to measure regional intramuscular temperature in the vastus medialis and triceps brachii muscles (Kenny et al. 2006). A flexible multi-sensor intramuscular temperature probe (Physitemp Instruments, Clifton. New Jersey, USA, model IT-17:4), consisting of four sensors located at 0, 15, 30, and 45 mm from the probe tip, was used along with an ultrasound guidance system to accurately place the sensors on the required depths in each of the vastus medialis and triceps brachii muscles. The temperature probe was inserted into the vastus medialis midway between the anterior superior iliac spine and the patella at an average depth of 47.9 mm, such that the tip was ~ 10 mm from the femur bone and the deep femoral artery. Another temperature probe was inserted into the triceps brachii midway between the scapula and the ulna to an average depth of 34.2 mm, with the tip ~ 10 mm from the humerus bone and the superior ulnar collateral artery. The subjects arrived in the laboratory at 8:00 a.m. and were seated in the upright position for 75 min at 25 °C before the temperature recordings began.

#### Study 2: Rectal temperature measurements during heat stress

For rectal temperature validation, we used three separate experimental studies, each representing different types of physical activity: (a) strenuous physical activity on a treadmill, (b) walking on a treadmill (Kazman et al. 2015), and (c) pedaling on a bicycle ergometer (Stolwijk 1971). *Study* 2a represents a new effort performed at the University of Connecticut, whereas *Study* 2b and *Study* 2c have been previously published.

##### *﻿Study* 2a

**Fig. 1:**
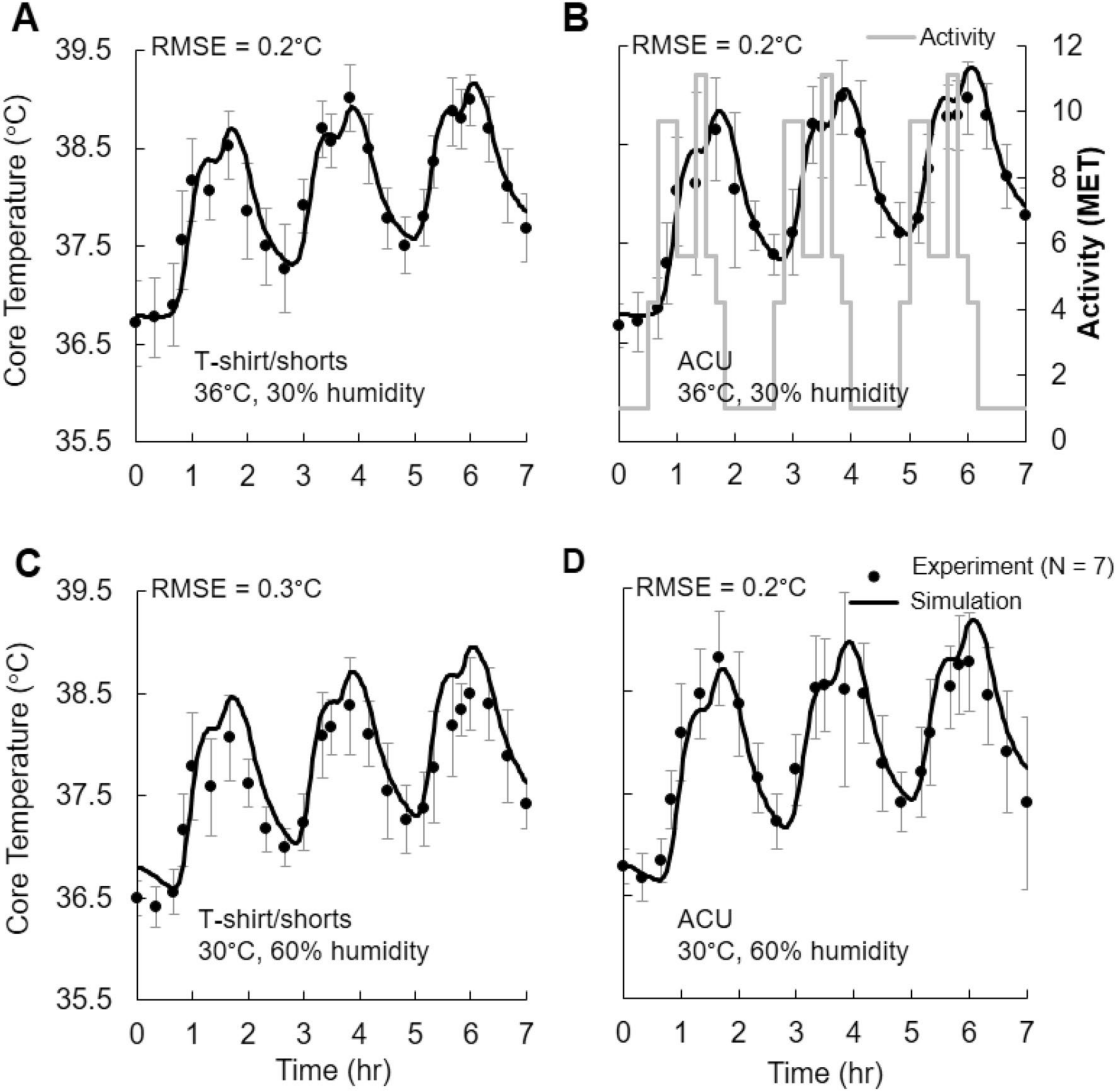
Model validation for *Study* 2a in which seven subjects perform three extraneous, time-varying exercise bouts under four different conditions. A rectal probe served to measure core temperature. The clothing and environmental conditions varied across the four exercise conditions: **a** T-shirt/shorts at 36 °C and 30% humidity, **b** active combat uniform (ACU) at 36 °C and 30% humidity, **c** T-shirt/shorts at 30 °C and 60% humidity, and **d** ACU at 30 °C and 60% humidity. Filled circles denote the mean experimental values (*N* = 7), while the vertical bars represent one standard error of the mean. *MET* metabolic equivalent of task, *RMSE* root mean squared error

Seven young healthy men participated in the study [mean age (standard deviation), 19.4 (1.1) years], in which for 7 h per session they exercised at various intensity levels on a treadmill inside an environmentally controlled chamber. Table 1 shows their anthropometric measurements. Each 7-h session started at ~ 8:30 a.m., with a 60-min rest period, after which subjects underwent three identical high-intensity exercise bouts, each lasting for 80 min followed by a 50-min rest period (Fig. 1b). Each subject repeated this exercise protocol for four sessions, each under different clothing and environmental conditions: at 36 °C and 30% relative humidity wearing T-shirt/shorts; at 36 °C and 30% relative humidity wearing an active combat uniform (ACU); at 30 °C and 60% relative humidity wearing T-shirt/shorts; and at 30 °C and 60% relative humidity wearing an ACU. Prior to data collection, we obtained oxygen consumption (VO_2_) values for each subject under similar activity levels, which we converted to metabolic equivalent task (MET) units [1 MET = VO_2_ (ml/min/kg)/3.5 (ml/min/kg)] and used as an input to the model. The Institutional Review Boards (IRBs) of the University of Connecticut (Storrs, Connecticut, USA) and the U.S. Army (Human Research Protection Office, U.S. Army Medical Research and Development Command, Frederick, Maryland, USA) approved the study. Each subject provided a written consent. For the duration of the study, subjects consumed fluid ad libitum. A fan installed in front of the subjects provided wind at a speed of 2.5 m/s. A rectal probe (YSI 400 series probe, Measurement Specialties, Hampton, Virginia, USA; accuracy ± 0.1 °C) inserted 10–15 cm beyond the anal sphincter served to measure rectal temperature utilizing a continuous physiological monitoring system (Biopac Systems Incorporated, Santa Barbara, California, USA), with a sampling rate of 250 Hz.

**Table 1:** Anthropometric measurements for the seven men in *Study* 2a

Subject	Age (years)	Height (m)	Weight (kg)	VO_2_ max (ml.kg^−1^.min^−1^)
1	18	1.67	58.5	48.6
2	20	1.82	92.9	45.4
3	20	1.94	93.3	45.3
4	21	1.71	58.0	52.3
5	19	1.84	82.2	53.1
6	18	1.76	64.5	52.7
7	20	1.86	75.1	52.4

##### *Study﻿* 2b

Sixty subjects [42 men and 18 women, mean age (standard deviation) 27.0 (5.7) years] participated in a heat-tolerance test (Kazman et al. 2015), in which they walked for 2 hours at a rate of 5 km/h on a treadmill with a grade of 2%, in an environmental chamber maintained at 40 °C and 40% relative humidity, with the men wearing only shorts and the women wearing shorts and sports bra. The experiments were conducted during the morning hours (we assumed 9:00 a.m. for our simulations). The IRB of the Uniformed Services University (Bethesda, Maryland, USA) approved the study. Each subject provided a written consent before the study, after being informed about its purposes and procedures.

Subjects drank water ad libitum with an upper limit of 1 l/h. A rectal thermometer (MEAS Temperature Probe, Measurement Specialties, Dayton, Ohio, USA; accuracy ± 0.1 °C) inserted 10 cm beyond the anal sphincter served to measure rectal temperature, every 15 s.

##### *Study﻿* 2c

Three minimally dressed men (in shorts) underwent a bicycle ergometer study at 30 °C, 30% relative humidity, and an air velocity of 0.1 m/s (Stolwijk 1971). They completed three 30-min exercise bouts of increasing intensity (2.6, 4.9, and 8.4 MET), each of which was followed by a 30-min rest period. The authors did not report the time of day for the experiments (we assumed 9:00 a.m. for our simulations).

### Computational model

**Fig. 2:**
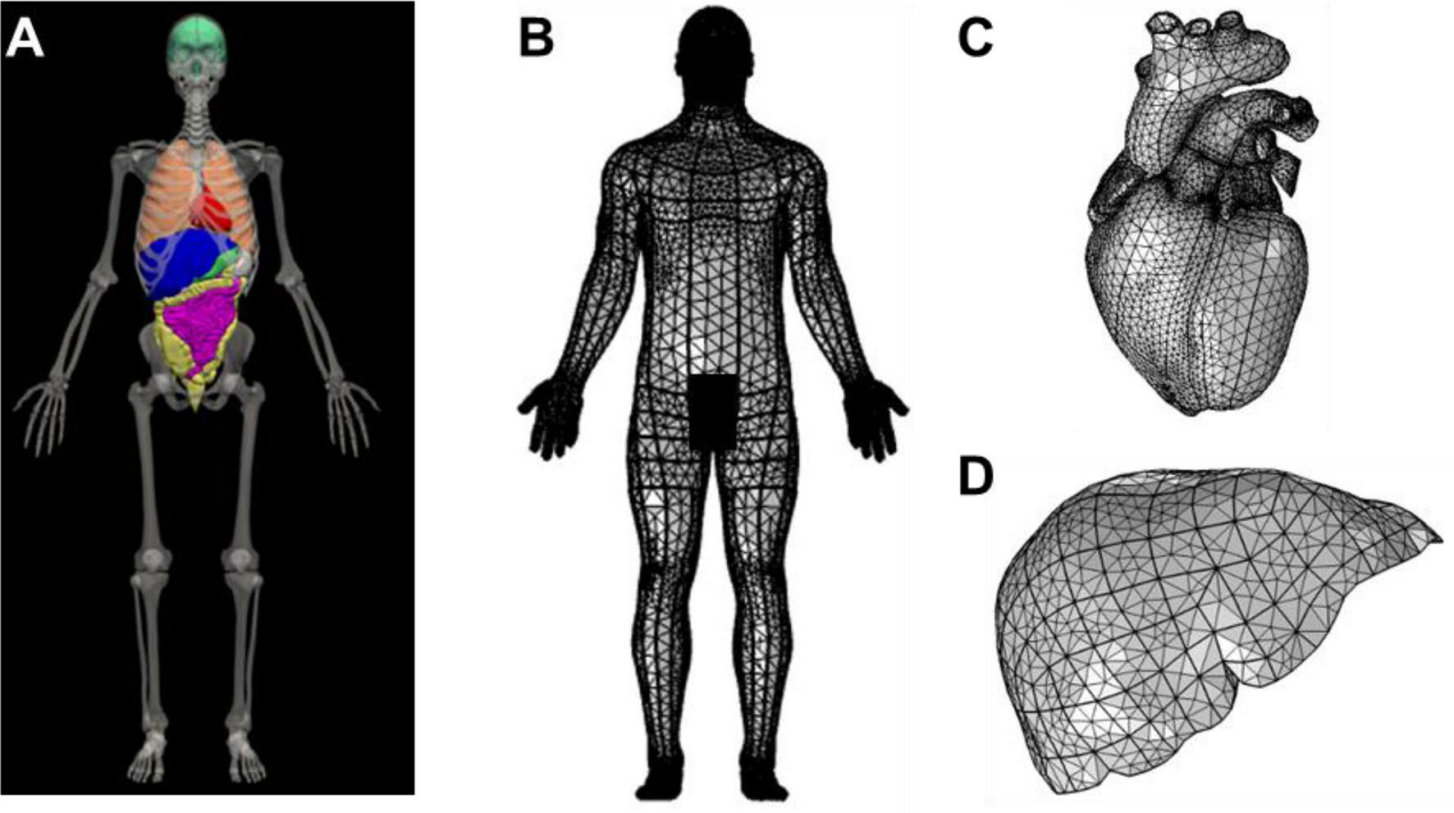
Skeletal system and internal organs of a 50th percentile U.S. male, acquired from Zygote (**a**). Finite element mesh generated for the whole body (**b**), heart (**c**), and liver (**d**)

**Fig. 3:**
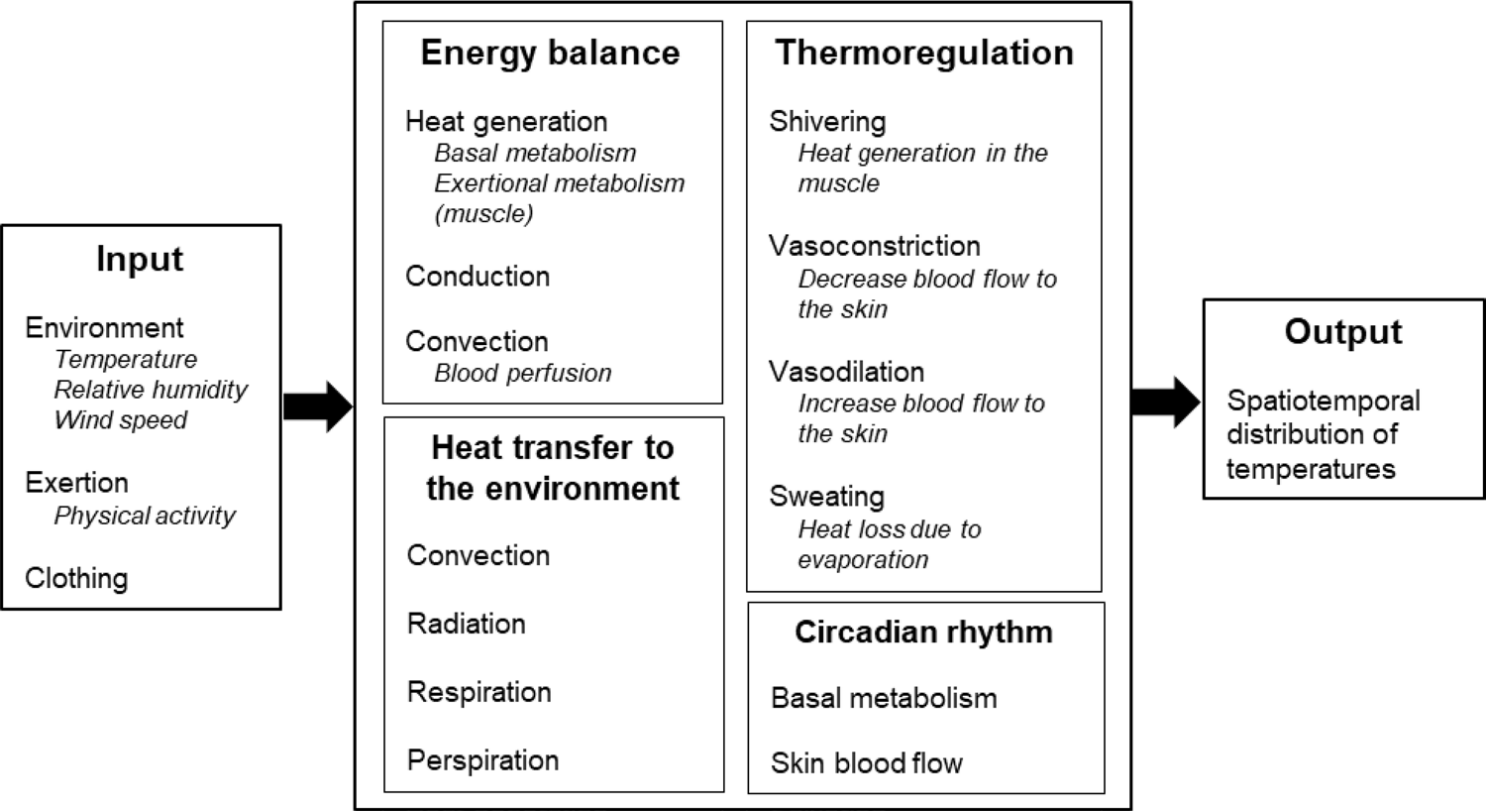
Overview of the thermoregulatory model of the 3-D virtual human. The thermoregulatory model takes environmental conditions (i.e., atmospheric temperature, relative humidity, and wind speed), physical activity, and clothing as inputs and predicts the spatiotemporal distribution of temperatures in the entire virtual human. The model accounts for the transfer of energy within the body, heat transfer from the body to the environment, the major thermoregulatory mechanisms, and the day-night cycle of temperature changes due to circadian-rhythm effects

To simulate the response of environmental and exertional heat-stress conditions in humans, we developed a 3-D thermoregulatory virtual human FE model, which realistically represented the anatomy of a 50th percentile U.S. male (Fig. 2). The model takes environmental conditions (i.e., atmospheric temperature, relative humidity, and wind speed), physical activity, and clothing as inputs and predicts the spatiotemporal distribution of temperatures in the entire virtual human as an output. As shown in Fig. 3, the model accounts for the transfer of energy within the body, heat transfer to the environment, the major thermoregulatory mechanisms, and the day–night cycle of temperature changes due to circadian-rhythm effects. To represent the macroscopic energy balance within the body, we used the Pennes bioheat transfer equation, wherein we modeled heat generated in the body due to basal metabolism and physical activity, heat conduction, and heat convection through blood perfusion. We accounted for heat transfer from the body to the environment by representing convection, radiation, respiration, and perspiration mechanisms, while considering the thermoregulatory mechanisms of shivering, sweating, vasoconstriction, and vasodilation. In addition, we accounted for circadian-induced temperature variations by changing the basal metabolic heat generation and skin blood flow as a function of time of day.

#### Anatomical model of a virtual human

Our virtual human model is based on an anatomically detailed geometry of a 50th percentile U.S. male (Solid 3D Male Model, Zygote, American Fork, Utah, USA) (Pheasant et al. 2005; Roebuck 1995; Military Handbook 1991). The geometry’s key body characteristics include weight 83.8 kg, height 175 cm, body fat content 15%, and body surface area 1.9 m^2^. This geometry, in the ParaSolid format, comprised of all of the major organs of a human, including the brain, heart, lungs, liver, intestines, as well as the skeletal system. Using MeshLab (Cignoni et al. 2008), we modified the geometries of the small and large intestines to rectify imperfections, such as intersecting boundaries and distortion of surfaces. Next, we imported the geometry of the virtual human into a FE software package, COMSOL Multiphysics (COMSOL, Burlington, Massachusetts, USA), to develop the thermoregulatory model. In COMSOL, we added a thin layer of skin dermis (1.0 mm) and epidermis (0.1 mm) of uniform thicknesses to the virtual human model (Lee and Hwang 2002).

#### Energy balance

In our thermoregulatory model, we represented the heat transfer occurring within the body using the bioheat transfer equation of Pennes (Fiala et al. 2012; Pennes 1948; Wissler 1998), wherein the dynamic change in temperature* T* within a tissue is assumed to be dependent on heat conduction, convective heat transfer via blood perfusion, and heat generation due to metabolic activity* Q*_*m*_, and is represented by

1 $$\rho c_{p} \frac{\partial T}{{\partial t}} = \nabla .\left( {k\nabla T} \right) + \rho_{b} c_{p,b} \omega_{b} \left( {T_{b} - T} \right) + Q_{m} ,$$

where* ρ*, ${{c}}_{{p}}$, and ${{k}}$ denote the density, specific heat capacity, and thermal conductivity of the tissue, respectively, $\rho_{b}$ and ${{c}}_{{p,b}}$ represent the density and specific heat capacity of the blood, respectively, $\omega_{b}$ represents the perfusion rate of the blood in a tissue, and ${{T}}_{{b}}$ denotes the arterial blood temperature. We obtained the thermophysical properties, such as density, specific heat capacity, and perfusion, of tissue and blood from the literature (Table S1 in the online Supplementary Material) (Fiala et al. 1998; Hasgall et al. 2018).

*Metabolic heat generation* Following the works of Fiala et al. (Fiala et al. 1998, 1999, 2012), we represented the metabolic heat generated in tissue* Q*_m_ as the sum of the basal metabolism* Q*_*m*0_ (Table S1 in the online Supplementary Material) and additional heat $\Delta Q_{m}$ generated due to changes in the basal metabolism, exertion, and shivering* Q*_shivering_. This additional heat in the tissue is represented as

2 $$\Delta Q_{m} = Q_{m0} \left[ {2^{{\left( {\frac{{T {-} T_{0} }}{10}} \right)}} - 1} \right] + \frac{{\partial \left( {a_{m,w} H} \right)}}{\partial V} + Q_{{{\text{shivering}}}} ,$$

where *T*_0_ denotes the temperature of the tissue at its thermoneutral state, *H* denotes the internal whole-body workload, *a*_*m,w*_ represents the distribution coefficient of the workload in the tissue, and *V* denotes the volume of the tissue. We assumed that heat generation from exertion and shivering occurs only in the muscle tissues. In our model, we represented the internal workload *H* as the difference between the actual overall heat-generation rate and the basal metabolism (Fiala et al. 1999), by the following equation:

3 $$H = M_{{{\text{bas}},0}} \left[ {\frac{{{\text{act}}}}{{{\text{act}}_{{{\text{bas}}}} }}\left( {1{-} \eta } \right) {-} 1} \right],$$

where *M*_bas,0_ denotes the basal metabolic value of a resting individual in a reclining position, the ratio act/act_bas_ denotes the ratio of the intensity of the activity level of an individual (measured in MET) with respect to the individual’s basal activity level at rest, and* η* refers to the efficiency by which energy generated by the body is converted into useful work, which is set to zero for low-activity levels (act < 1.6 MET) and is given as $\eta = 0.2 \tanh \left( {0.39 {\text{act}} {-} 0.60} \right)$ (Fiala et al. 1999; Pokorný et al. 2017) for normal and high-activity levels. Table 2 lists the activity levels corresponding to the different studies considered in our work. Details regarding the heat generated due to shivering, which occurs only during cold stress, are provided in the online Supplementary Material (Eq. S11).

**Table 2:** Boundary conditions and input parameters

Study	Ambient temperature (°C)	Relative humidity (%)	Wind speed (m/s)	Clothing	Metabolic rate (MET)
1					
Thermoneutral	30	40	0.05	Shorts	0.8
2a					
Very hot and dry, T-shirt	36	30	2.50	T-shirt/shorts	See Fig. 1b
Very hot and dry, ACU	36	30		ACU	
Hot and humid, T-shirt	30	60		T-shirt/shorts	
Hot and humid, ACU	30	60		ACU	
2b					
Heat-tolerance test	40	40	0.30	Shorts	4.0
2c					
Bicycle ergometer	30	30	0.10	Shorts	See Fig. 6b

*Blood perfusion in a tissue* Similar to prior studies (Fiala et al. 1999, 2012), we assumed that the heat transfer between the blood and tissue, defined by the term $\rho_{b} c_{p,b} \omega_{b} \left( {T_{b} {-} T} \right)$ in Eq. 1, occurs in the capillary beds. In addition, we assumed that the temperature of the blood in the small arteries is the same as the average temperature of the blood in the heart, the temperature of venous blood flowing out of an organ is equal to the temperature of the tissue, and there is no heat loss from the arterial blood to the venous blood. We also considered that blood perfusion rate in a tissue $\omega_{b}$, the arterial blood temperature *T*_*b*_, and the tissue temperature vary according to the external boundary conditions and the exertional heat stress.

During a thermoneutral state, tissues are perfused at basal perfusion rates (i.e., $\omega_{b} = \omega_{b,0}$; Table S1 in the online Supplementary Material). However, during a non-neutral thermal state as well as during exertion, the perfusion rate will be higher than the basal perfusion rate. This change in the basal perfusion rate $\Delta \omega_{b}$ of a tissue is represented in our model as

4 $$\Delta \omega_{b} = 0.932 \times \left[ {Q_{m0} \left( {2^{{\frac{{\left( {T - T_{0} } \right)}}{10}}} - 1} \right) + \frac{{\partial \left( {a_{m,w} H} \right)}}{\partial V} + Q_{{{\text{shivering}}}} } \right].$$

During the simulation, we calculated the arterial blood temperature *T*_*b*_ for the current time step using the resultant blood temperature (i.e., after mixing of venous blood from all organs) from the previous time step, using the following relationship:

5 $$T_{b } = \frac{{\mathop \smallint \nolimits_{V}^{{}} \omega_{b} T {\text{dV}}}}{{\mathop \smallint \nolimits_{V}^{{}} \omega_{b} {\text{dV}}}} .$$

#### Thermoregulatory mechanisms

In the virtual human model, we represented four thermoregulatory mechanisms of the central nervous systems, sweating, shivering, and vasodilation (increase) as well as vasoconstriction (decrease) of skin blood flow (Fiala et al. 2012). Details on the thermoregulatory mechanisms that influence heat generation and heat transfer within the body and from the body to the environment are provided in the online Supplementary Material.

#### Circadian rhythm

We accounted for the effect of circadian rhythm in both the metabolic heat generation and the skin blood flow, by allowing these quantities to vary sinusoidally so that metabolism is highest around noon and lowest during the late night (Kräuchi and Wirz-Justice 1994), while skin blood flow is highest at night and lowest during noon (Smolander et al. 1993). We used the following equations to model the circadian function for metabolic heat generation *C*_met_ and skin blood flow *C*_sbf_ as a function of time t (in seconds):

6 $$C_{{{\text{met}}}} = 1 + 0.115 \sin \left( {\frac{2\pi t}{{24 \times 60 \times 60}}} \right)$$

7 $$C_{{\text{sbf }}} = 1 {-} 0.200 \sin \left( {\frac{2\pi t}{{24 \times 60 \times 60}}} \right).$$

We multiply *C*_met_ by the corresponding tissue’s basal metabolism (*Q*_*m*,0_) and *C*_sbf_ by the basal value of the skin blood perfusion (*ω*_*b*,0_) to modulate them over a 24-h period.

#### Boundary conditions

*Heat transfer from unclothed surfaces* The body exchanges heat with the surroundings through convection and radiation. Heat transfer via convection is described as

8 $${-}k\frac{{\partial T_{{{\text{sk}}}} }}{\partial n} = h_{c} \left( {T_{{{\text{sk}}}} {-} T_{{{\text{amb}}}} } \right),$$

where ${{T}}_{{\text{s}}{\text{k}}}$ denotes the temperature at the surface of the skin,* n* indicates the direction normal to the surface, ${{T}}_{\text{amb}}$ represents the temperature of the surrounding air, and ${{h}}_{{c}}$ refers to the convective heat-transfer coefficient. This coefficient depends on the air velocity and is given by the following equation (Fiala et al. 1998):

9 $$h_{c} = \sqrt {a_{{{\text{nat}}}} \sqrt {T_{{\text{sk }}} {-} T_{{{\text{amb}}}} } + a_{{{\text{frc}}}} v_{a} + a_{{{\text{mix}}}} } ,$$

where ${{v}}_{{a}}$ denotes the air velocity and the coefficients ${{a}}_{\text{nat}}$, ${{a}}_{\text{frc}}$, and ${{a}}_{\text{mix}}$ denote the regression coefficients obtained from Fiala et al. (Fiala et al. 1998). Table 2 lists the ambient temperature and air speeds for the different studies. Heat transfer due to radiation is described as

10 $${-}k\frac{{\partial T_{{{\text{sk}}}} }}{\partial n} = \varepsilon \sigma \psi \left( {T_{{{\text{sk}}}}^{4} {-} T_{{{\text{sr}},m}}^{4} } \right),$$

where $\varepsilon ,\,\sigma ,\,\psi$, and *T*_sr,m_ denote the emissivity (Fiala et al. 1998), the Stefan–Boltzmann constant, the view factor, and the mean radiant temperature, respectively. For our calculations, we obtained the corresponding view factors of the various body sectors from Fiala et al. (Fiala et al. 1998) and assumed that the mean radiant temperature is equal to the ambient temperature. After linearizing Eq. 10 and simplifying it, we obtained

11 $${-}k\frac{{\partial T_{{{\text{sk}}}} }}{\partial n} = h_{r} \left( {T_{{{\text{sk}}}} {-} T_{{{\text{amb}}}} } \right),$$

where $h_{r} = \left[ {\varepsilon \sigma \psi \left( {T_{{{\text{sf}}}} + T_{{{\text{amb}}}} } \right)} \right]$ (${{T}}_{\text{sf}}^{2}$ + ${{T}}_{\text{amb}}^{2}$)] denotes the radiative heat-transfer coefficient as a function of the ambient temperature.

*Heat transfer from clothed surfaces* The clothing layer provides insulation to the body and influences the heat loss from the body surface. We represented the heat transfer between the skin surface and the external environment through the clothing layer, using the following equation (Fiala et al. 1998):

12 $${-}k\frac{{\partial T_{{{\text{sk}}}} }}{\partial n} = U_{\text{cl}}^{*} \left( {T_{{{\text{sk}}}} {-} T_{{{\text{amb}}}} } \right),$$

where ${{U}}_{\text{cl}}^{*}$ denotes the local effective heat-transfer coefficient. The local effective heat-transfer coefficient ${{U}}_{\text{cl}}^{*}$ (Eq. 12) accounts for both the convective and radiative heat transfer occurring between the skin and the external environment as well as the effect of clothing, as follows:

13 $$U_{\text{cl} }^{*} = \frac{1}{{\mathop \sum \nolimits_{m = 1}^{M} (I_{cl}^{*} )_{m} + \frac{1}{{f_{cl}^{*} \left( {h_{c} + h_{r} } \right)}}}},$$

where ${\left({{I}}_{\text{cl}}^{*}\right)}_{{m}}$ denotes the local thermal resistance of the *m*th clothing layer, ${\text{f}}_{\text{cl}}^{*}$ indicates the local clothing factor of the outermost clothing, and M denotes the total number of clothing layers. Table 2 lists the different clothing ensembles used in the various studies with the corresponding clothing parameters indicated in Table 3. The effect of clothing on the evaporation of sweat from the body surface is described in the online Supplementary Material.

**Table 3:** Clothing parameters used for simulations

Clothing	Local thermal resistance	Local moisture permeability index	Local clothing area factor	Local sensible effective heat-transfer coefficient	Local evaporative heat-transfer coefficient
	${{I}}_{\text{cl}}^{*}$	${{i}}_{\text{cl}}^{*}$	${{f}}_{\text{cl}}^{*}$	${{U}}_{\text{cl }}^{*}$	$U_{{E{\text{,cl}}}}^{*}$
	(clo)^a^	(1)	(1)	(W.m^−2^.K^−1^)	(W.m^−2^.Pa^−1^)
Shorts (Curlee 2004)	0.54	0.5	1.24	–	–
T-shirt (Curlee 2004)	0.59	0.7	1.05	–	–
ACU	–	–	–	7.5^b^	0.06^b^
Foot wear(Curlee 2004)	2.73	0.0	1.43	–	–

^a^1 clo = 0.155 W^−1^.m^2^.K

^b^Values correspond to a wind speed of 2.5 m/s (Sennett et al. 2017)

*Respiration* We also accounted for the net heat loss or gain due to respiration by combining the latent heat exchange ${{E}}_{\text{rsp}}$ and the dry heat loss ${{C}}_{\text{rsp}}$, which were represented as (Fiala et al. 1998, 2012) follows:

14 $$E_{{{\text{rsp}}}} = 3.233 \times \mathop \smallint \limits_{V}^{{}} Q_{m} {\text{dV}}\left( {0.0277 {-} 6.5 \times 10^{ - 5} T_{{{\text{amb}}}} {-} 4.91 \times 10^{ - 6} p_{{{\text{amb}}}} } \right),$$

15 $$C_{{{\text{rsp}}}} = 1.44 \times 10^{ - 3} \mathop \smallint \limits_{V}^{{}} Q_{m} {\text{dV}}\left( {32.6 {-} 0.934 \times T_{{{\text{amb}}}} + 1.96 \times 10^{ - 4} p_{{{\text{amb}}}} } \right),$$

where *p*_amb_ denotes the partial vapor pressure of the surrounding air. We assumed the net heat transfer due to respiration (*E*_rsp_ + *C*_rsp_) to be distributed in the lungs, upper respiratory region, and neck, as follows: lungs 30%; trachea and muscle band 25%; and inner and outer facial muscles 45% (Fiala et al. 1998).

#### FE meshing and model initialization

*FE meshing of the virtual human* Using the adaptive meshing algorithm in COMSOL, we meshed the geometry of the virtual human using 4.4 million linear elements. Next, for each element of the FE mesh, we assigned the appropriate energy balance equation (Sect. “Energy balance”), thermoregulatory mechanisms (Sect. “Thermoregulatory mechanism”), and corresponding boundary conditions (Sect. “Boundary conditions”). Then, using COMSOL, we solved the above equations with a time step of 2.5 min to obtain the spatially and temporally varying 3-D temperature distributions in the virtual human for different environmental and exertional heat-stress conditions. We invoked the *nojac* operator in COMSOL in all our simulations, and identified the optimum number of elements and the optimal time step for all simulations by performing mesh-convergence and time-step convergence studies, respectively.

*Initialization of the FE model* To initialize the virtual human model before each study, we determined the initial temperature distribution in the entire body by performing a steady-state FE simulation without any physical activity.

## Results

### Mesh- and time-step-convergence studies

We performed a mesh-convergence study on the virtual human model using four different mesh sizes corresponding to 2.0, 3.5, 4.4, and 5.2 million elements. In this study, we simulated steady state for a boundary condition corresponding to the thermoneutral state (i.e., 30 °C and 40% relative humidity with T-shirt/shorts and without any physical activity) and monitored core body temperature at the rectum. When the number of elements were increased from 2.0 to 4.4 million, the core body temperature increased from 36.8 to 36.9 °C. However, we did not observe any difference in the core body temperature between the FE model with 4.4 million and 5.2 million elements. Hence, we selected the FE model with 4.4 million elements for all subsequent analysis.

We also performed a time-step convergence study, using the FE model with 4.4 million elements, to determine the optimal time step for our FE analyses. We predicted core body temperature as we simulated a strenuous physical activity at an ambient temperature of 30 °C and 40% relative humidity with T-shirt/shorts (*Study* 2a, Fig. 1a) using four different time steps: 10.00, 5.00, 2.50, and 1.25 min. When compared to a 1.25-min time step, the root mean squared error (RMSE) of the core temperature time profile predicted with time steps of 10.00 and 5.00 min was 0.10 °C. However, the predicted temperature profiles obtained with time steps of 1.25 and 2.50 min were nearly identical (RMSE = 0.0 °C). Hence, we selected a time step of 2.50 min to reduce the computational time of our analysis without losing prediction accuracy. For simulating the results of this 7-h study, it took approximately 6 h of CPU time on a 3.4 GHz 4-core Intel-i7 Workstation with 64 GB RAM.

### Model validation

We validated our virtual human FE model by comparing the model predictions with experimental measurements of organ and muscle temperatures under normal resting conditions, as well as rectal body temperature under heat-stress conditions.

#### Organ temperature validation

**Fig. 4:**
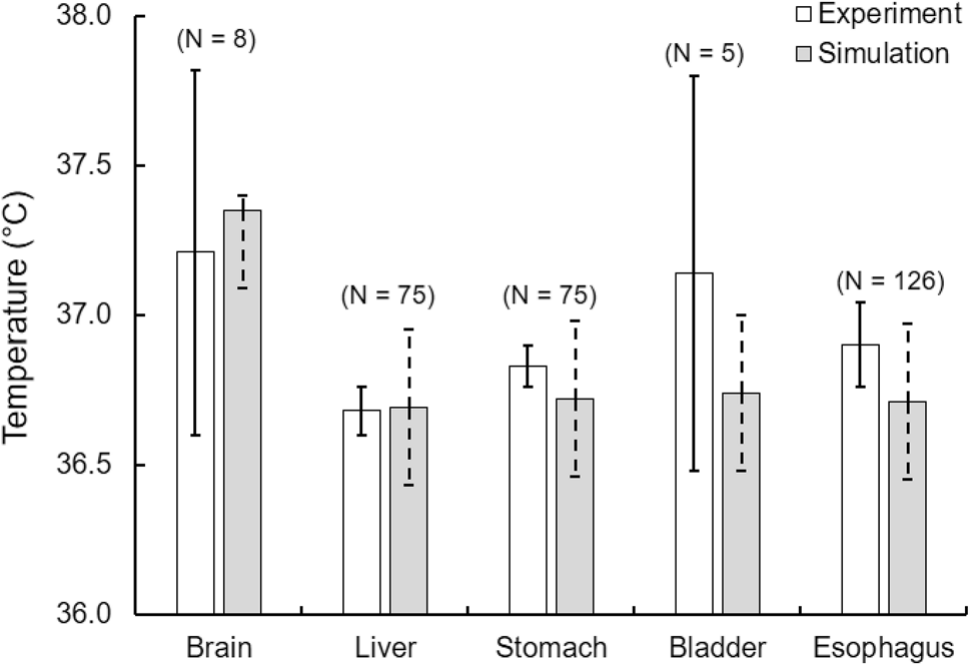
Model validation based on measured temperatures of key organs under normal resting conditions (assumed to be at 30 °C and 40% humidity). Mean experimental values (white bars) collected from *Studies* 1a–d, with N denoting the number of subjects used in each study. The predicted temperatures are represented by gray bars, for each organ. The predicted temperature of the brain was evaluated at 6:00 p.m., based on reported measurement times. For the liver, stomach, bladder, and esophagus, we predicted their values for 10:00 a.m. The dotted line for the predictions indicates the minimum and maximum temperature arising from circadian effects within a 4-h period, i.e., between 4:00 p.m. and 8:00 p.m. for the brain, and between 8:00 a.m. and 12:00 p.m. for the other organs. The solid line for experimental values represents the 95% confidence interval of the mean

We compared the model-predicted volume-averaged temperature of various organs (brain, liver, stomach, bladder, and esophagus) against averaged experimental values from multiple separate studies (Sect. “Study 1: Organ and muscle temperature measurements during normal resting conditions” *Studies* 1a–d). We assumed thermoneutral conditions (30 °C and 40% humidity) with subjects wearing shorts and reclining (act = 0.8 MET) whenever the original study did not report the boundary conditions for organ-temperature validation. For each organ, we found that the predicted volume-averaged temperatures lay between 0.0 and 0.4 °C of the experimental values (Fig. 4). Because the organ temperature changes as a function of time of day and the precise time of the measurements was not always provided, we used the available information to estimate the measurement time for our predictions. In addition, we report predicted temperatures at 2 hours before and 2 hours after the estimated measurement time to provide a prediction “error bound” around the estimated time.

For the brain, the temperature measurements took place in the evening between 5:00 and 7:00 p.m. Accordingly, we computed the brain temperature from the model at 6:00 p.m. To compare the model-predicted brain temperature against the measured data, we computed the volume-averaged temperature of the brain cerebrum as an approximation for the VOI in the frontal cortex used in the experiment. The predicted temperature compared well with the measurements, yielding an error of less than 0.2 °C (Fig. 4).

Measurements for the liver and the stomach in *Study* 1b took place in the morning. Therefore, we evaluated their temperatures at 10:00 a.m., and indicated their variation arising from circadian effects between 8:00 a.m. and 12:00 p.m. Here again, we observed a good match between the FE predictions and the experimental values for both organs, with prediction errors of less than 0.2 °C (Fig. 4).

For the bladder and the esophagus (*Study* 1c and *Study* 1d, respectively), due to the lack of information on the measurement time, we assumed that the measurements took place in the morning and evaluated the temperatures at 10:00 a.m. For both the bladder and the esophagus, we observed that the range of the FE predictions around 10:00 a.m. (± 2 h) to be within the 95% confidence interval of the measured values, with prediction errors of less than 0.4 °C (Fig. 4).

#### Muscle temperature validation

For muscle temperature validation, we compared the model-predicted temperature at various depths inside the vastus medialis and triceps brachii muscle groups under normal resting conditions (*Study* 1e).

**Fig. 5:**
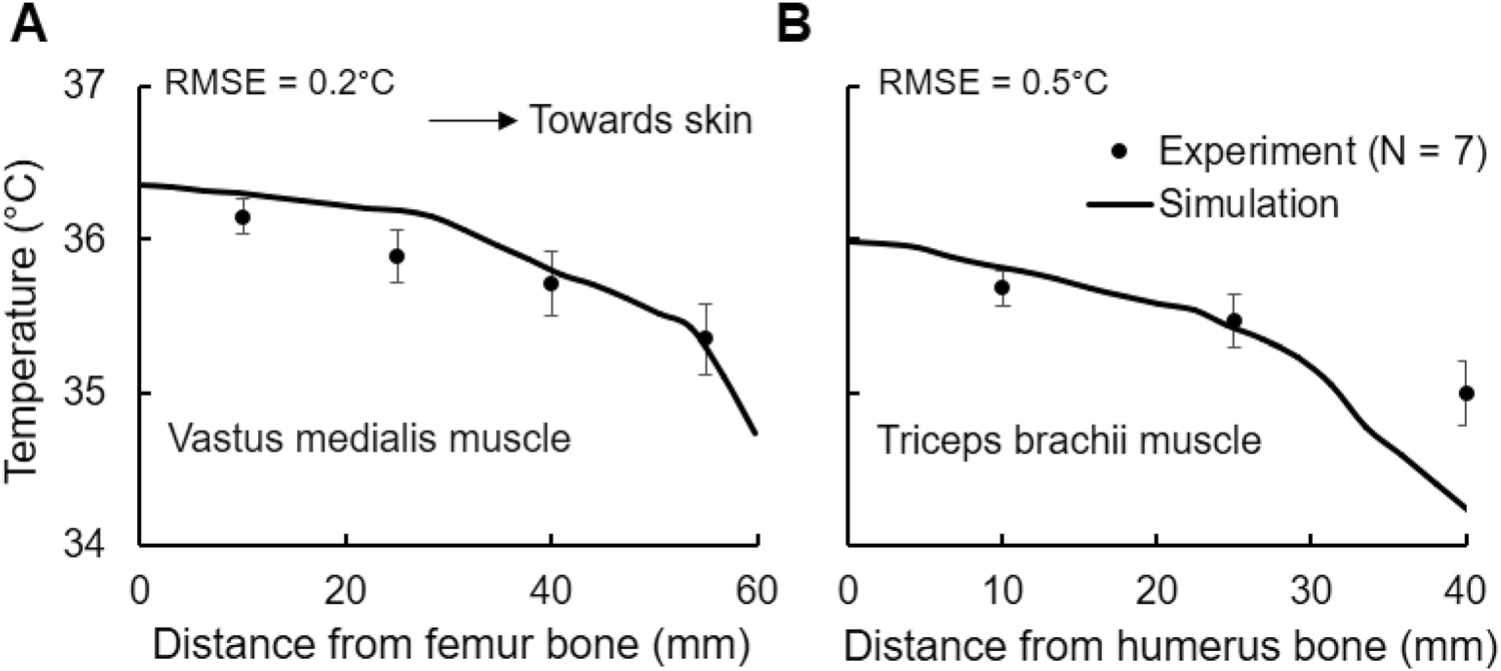
Model validation using muscle temperature measurements at various depths inside **a** the vastus medialis (thigh) and **b** the triceps brachii (upper arm) in which seven male subjects sat at 25 °C for 75 min, starting at 8:00 a.m. (*Study* 1e). The filled circles represent muscle temperature measurements at various distances from the bone averaged across the subjects at each site, and the gray vertical bars represent the 95% confidence interval of the mean. The solid continuous line indicates the model predictions

In the study and in our simulations, the intramuscular temperatures decreased as we moved away from the bone towards the skin (Fig. 5). In the vastus medialis, the measured temperature dropped from 36.2 to 35.4 °C with increasing distances from the femur bone, whereas the FE model captured this trend and predicted the measured values at various depths well (Fig. 5a, RMSE = 0.2 °C). Similarly, the measured temperature for the triceps brachii decreased from 35.7 to 35.0 °C as the distance from the humerus bone increased (Fig. 5b, RMSE = 0.5 °C). The relatively large RMSE observed for the triceps brachii resulted from an under-prediction of the temperature at the 40-mm site, which is very close to the skin surface. Any localized differences in fat thickness (insulation) and the positioning of the temperature sensor next to the skin can have a large impact in the measured temperature, which can possibly explain the discrepancy between the predicted and measured muscle temperature near the skin. Nevertheless, the model predictions at the other two sites (10 and 25 mm from the bone) in the triceps brachii were within the 95% confidence interval of the mean values, indicating a good agreement between the model predictions and the measured data for muscle temperatures.

#### Rectal temperature validation

We compared the rectal temperature for three separate heat-stress studies: (1) *Study* 2a: strenuous physical activity on a treadmill under four different clothing/environmental conditions, (2) *Study* 2b: walking on a treadmill, and (3) *Study* 2c: pedaling on a bicycle ergometer. We used the FE predictions of core body temperature at the center of a transverse cross-sectional plane of the rectal cavity at a depth of 10 cm from the anal sphincter to represent the predicted rectal temperature, which we compared against the experimental measurements obtained with a rectal probe.

For *Study* 2a, we observed a good match between the FE predictions and the experimental measurements for each of the four conditions (Fig. 1, RMSE values ranged from 0.2 °C to 0.3 °C). For each condition, our FE model was able to capture the rise in core temperature during each of the three exercise bouts (exercise intensity represented by gray traces in Fig. 1b) as well as the drop in temperature during the 50-min rest period (i.e., activity = 1 MET) between the bouts. The predicted core temperature values consistently peaked during the third exercise bout for each of the four experimental conditions, with a maximum temperature rise of ~ 2.5 °C from the pre-activity level. Surprisingly, we did not observe any meaningful differences in the temporal variation of the core body temperature due to different clothing (i.e., T-shirt/shorts vs. ACU) or environmental conditions (i.e., 30 °C, 60% humidity vs. 36 °C, 30% humidity), in the experimental data or in the model predictions.

**Fig. 6:**
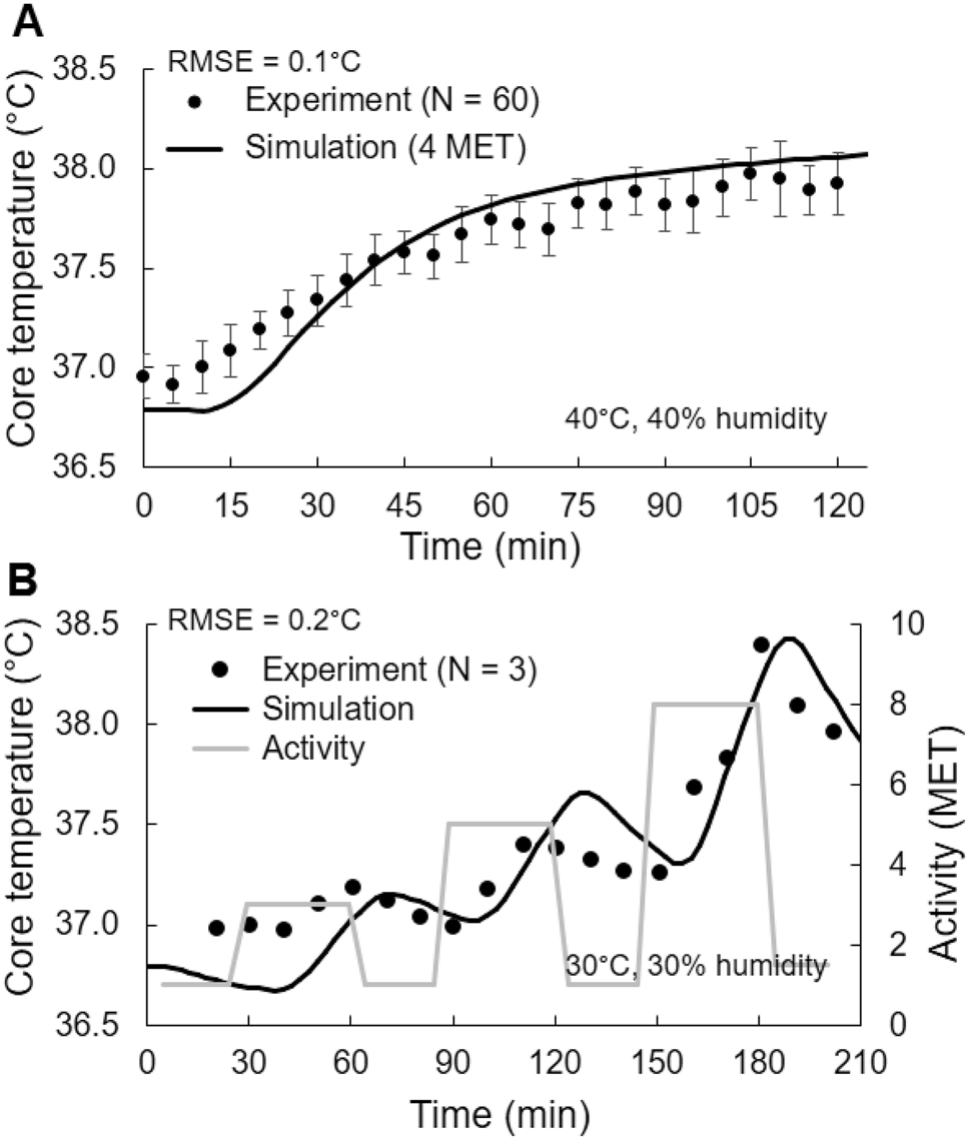
Model validation for *Study* 2b **a** in which 60 subjects walked on a treadmill at 5 km/h for 120 min and for *Study* 2c **b** in which three subjects pedaled on a bicycle ergometer at varying intensity for 210 min. Filled circles denote the mean experimental values, while the vertical bars in A represent one standard error of the mean. *RMSE* root mean squared error

Similar to *Study* 2a, we observed a close match between the predicted and measured core body temperature for *Study* 2b (Fig. 6a, RMSE = 0.1 °C) and *Study* 2c (Fig. 6b, RMSE = 0.2 °C). As expected, in *Study* 2b the core temperature increased monotonically with time when the subjects walked at a constant intensity of 4 MET. In contrast, in *Study* 2c the core temperature increased with increasing physical activity and decreased during the rest periods (activity = 1 MET; the activity levels are represented as grey traces in Fig. 6b).

### Temperature variation within the rectal cavity during heat stress

**Fig. 7:**
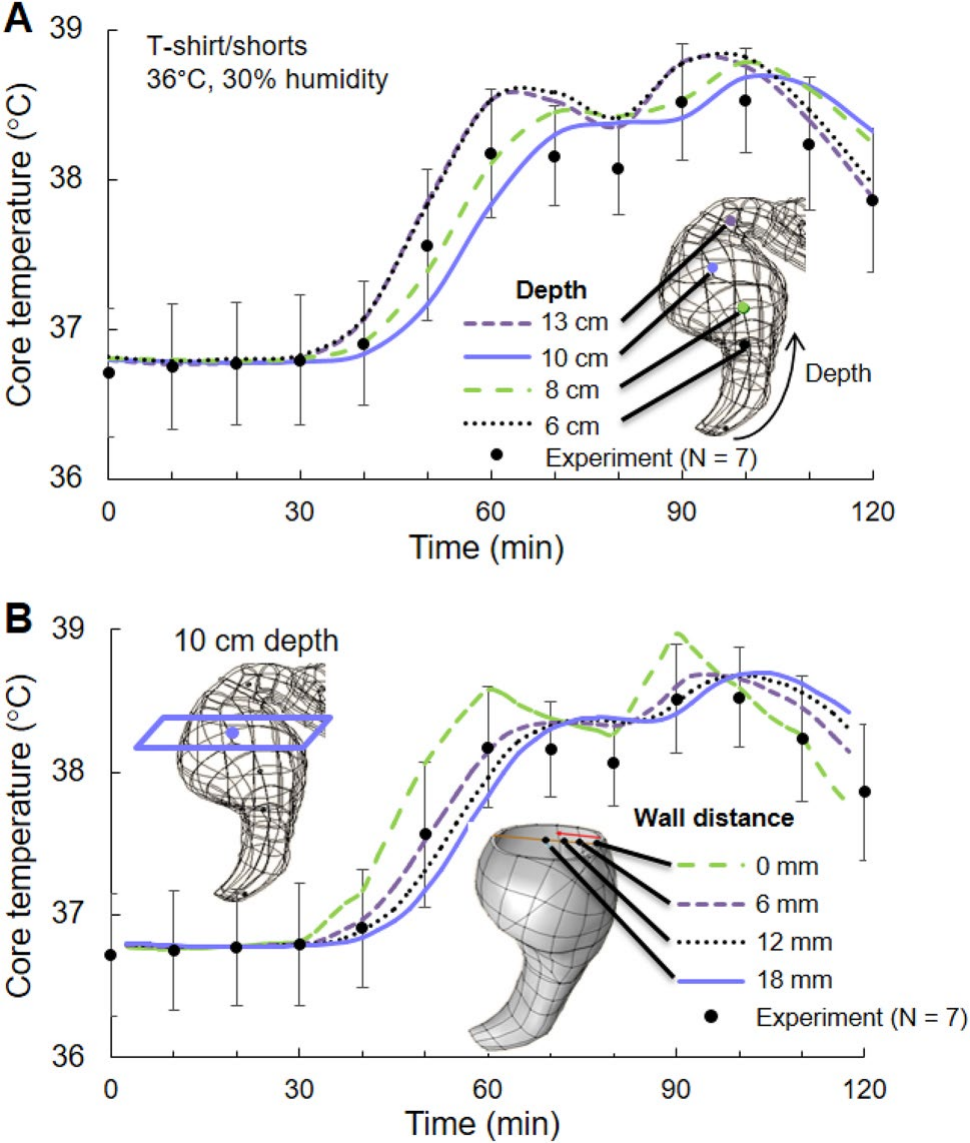
**a** Temporal distribution of core body temperature at multiple locations along the depth of the rectal cavity (6–13 cm). **b** Variation in core body temperature at the 10-cm depth into the rectal cavity as a function of distance from the cavity wall (0–18 mm). The simulated results are for the condition with T-shirt/shorts at 36 °C and 30% humidity in “*Study* 2a” (Fig. 1a). Filled circles denote the mean experimental values (*N* = 7), while the vertical bars represent one standard error of the mean

Using the virtual human model, we quantified the variation of temperature within the rectal cavity for the first exercise bout (duration of 120 min) in *Study* 2a with T-shirt/shorts at 36 °C and 30% humidity (corresponding to Fig. 1a). First, we determined the temporal distribution of temperature at the center of a transverse plane at depths of 6, 8, 10, and 13 cm from the anal sphincter (Fig. 7a). Then, for the transverse plane at the 10-cm depth, we computed the temperature at distances of 0, 6, 12, and 18 mm from the rectal wall (Fig. 7b). At depths of 6 and 13 cm, the core temperature rose quicker and attained higher values than those at depths of 8 and 10 cm (RMSE = 0.2 °C). The predictions at 0 mm from the rectal wall differed most from the experimental data (RMSE = 0.3 °C), whereas the predictions at distances of 6 mm (RMSE = 0.1 °C), 12 mm (RMSE = 0.2 °C), and 18 mm (RMSE = 0.2 °C) were closer to the measured data (Fig. 7b). The peak rectal temperature at the wall (i.e., at the 0 mm distance) was greater than the peak temperature at locations within the rectal wall by at least 0.2 °C.

### Predicted spatiotemporal temperature distributions in the human body during heat stress

**Fig. 8:**
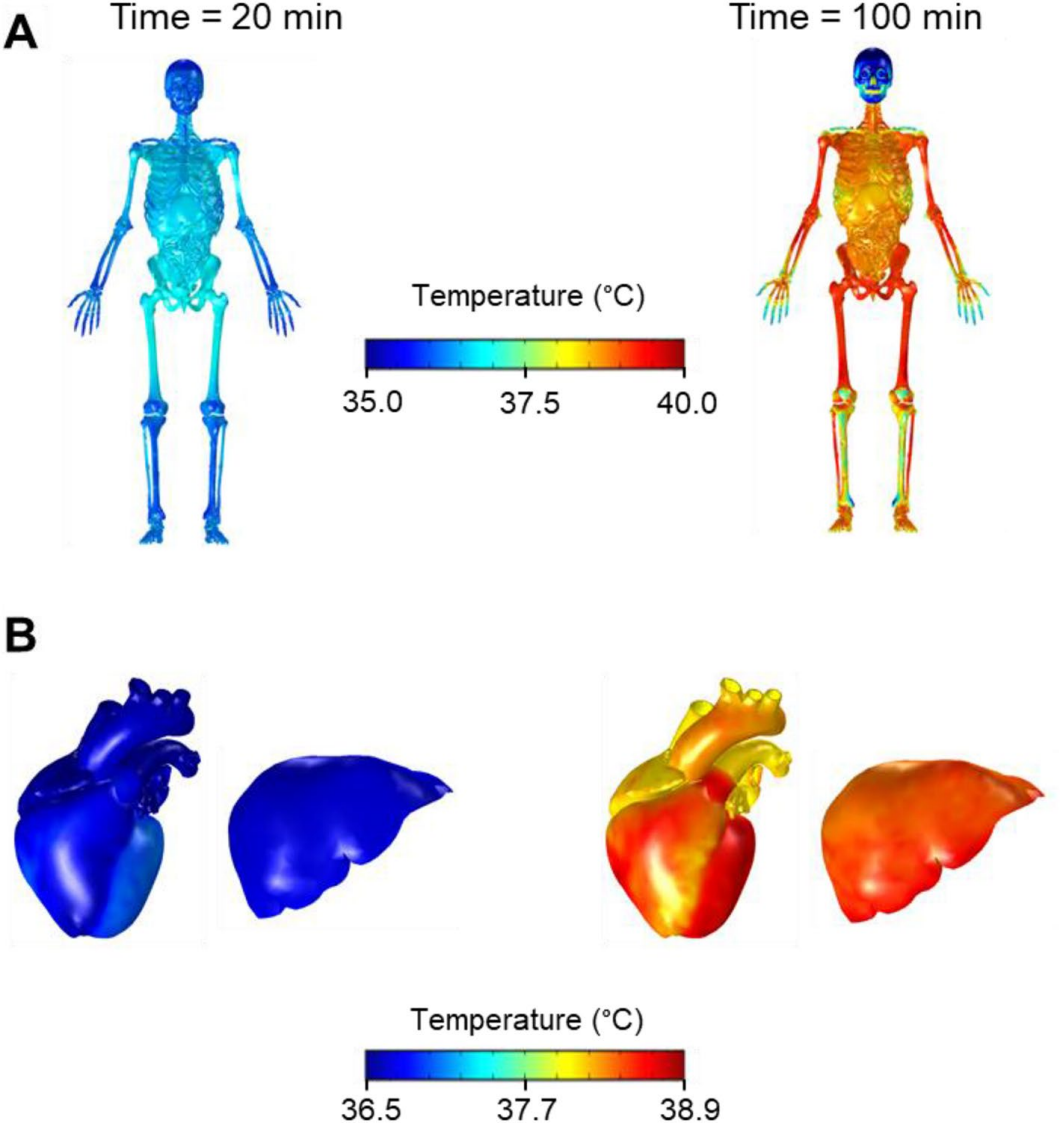
Predicted spatial temperature distribution for the whole body (**a**) and the liver and heart (**b**) for *Study* 2a with T-shirt/shorts at 36 °C and 30% humidity (Fig. 1a), at the thermoneutral condition (20 min before the start of the first exercise bout) and at 100 min into the study

Figure 8 shows the FE-model predictions of the spatial temperature distributions of the skeletal system and internal organs before the onset of *Study* 2a (Fig. 8, left panel) and at 100 min into the study, when the core temperature was at its peak (Fig. 8, right panel), for T-shirt/shorts, 36 °C and 30% relative humidity corresponding to the condition in Fig. 1a. The temperature around the abdomen and the thorax was approximately 36.7 °C at the onset of the study, but rose to 38.3 °C during the first exercise bout. The lower-abdomen and upper-leg regions showed the greatest increases in temperature because ~ 60% of the heat generated due to the exertional heat stress was concentrated in the muscles in these regions. The peripheral regions, such as the fingers and toes, showed the lowest temperature rises, as their large surface-to-volume ratios facilitated the transfer of heat to the environment through convection, radiation, and sweating. The skull was cooler than other body regions, as the muscles of the face and head do not generate heat during walking or running. We noted that heat conduction and internal convection (blood flow) were the most prominent modes of heat transfer by which the head absorbed the exertional heat generated by other regions of the body. Furthermore, unlike regions of the body containing muscles, subcutaneous fat, and skin, the skull was insulated from the environment only by the scalp.

### Variation in organ temperature during heat stress

**Fig. 9:**
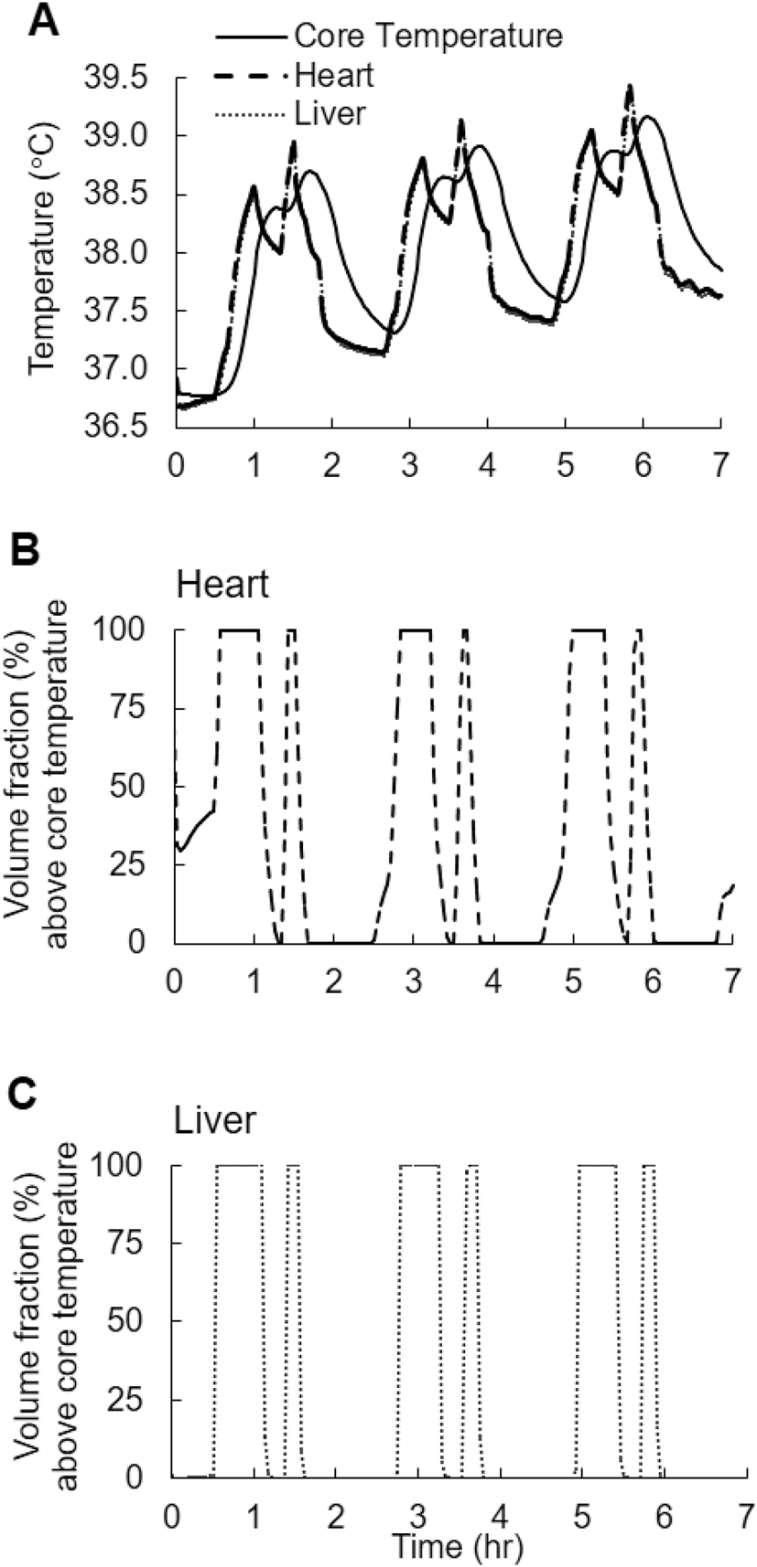
**a** Volume-averaged predicted temperatures in the heart and liver, and the rectum (core temperature) during the course of the exertional heat stress in *Study* 2a, corresponding to T-shirt/shorts at 36 °C and 30% humidity in Fig. 1a. Predicted volume fractions of the heart (**b)** and liver (**c)** with temperatures above those of the core

To study the response of the major organs of the body to the heat-stress condition corresponding to Fig. 1a, we used the virtual model to compute the volume-averaged peak temperatures (which provide an index of the overall heat load to an organ) and the peak local temperatures. The predicted volume-averaged, peak heart and liver temperatures obtained at 5.8 h into the study were 0.3 and 0.2 °C higher, respectively, than the predicted peak core temperature observed at 6.1 h (Fig. 9a). This slight delay is due to the thermal damping of the lumen in the rectal cavity (Sect. 3.3). Importantly, at 5.8 h into the study, 100% of the liver and the heart were above 38.5 °C, the lower-bound temperature for the onset of heat injury. Moreover, during the study, 100% of the volume of the heart (Fig. 9b) and the liver (Fig. 9c) were higher than the core temperature for at least 1.5 h out of the 7-h study. For all organs except the bones, the maximum volume-averaged temperature was higher than the peak core body temperature (39.2 °C), and the maximum local temperature at each organ was consistently higher than the peak core body temperature (Table 4).

**Table 4:** Predicted temperatures within different organs

Organ	Maximum volume-averaged temperature (°C)	Maximum local temperature (°C)
Bones	39.2	40.6
Brain	39.3	39.5
Core (Rectum)*	39.2	39.2
Gall bladder	39.4	39.8
Heart	39.4	39.8
Kidney	39.3	39.6
Liver	39.4	39.7
Small intestine	39.5	39.8
Spleen	39.4	39.7

*Predicted at a depth of 10 cm from the anal sphincter and 18 mm from the rectal wall

### Thermoeffector response to heat stress

**Fig. 10:**
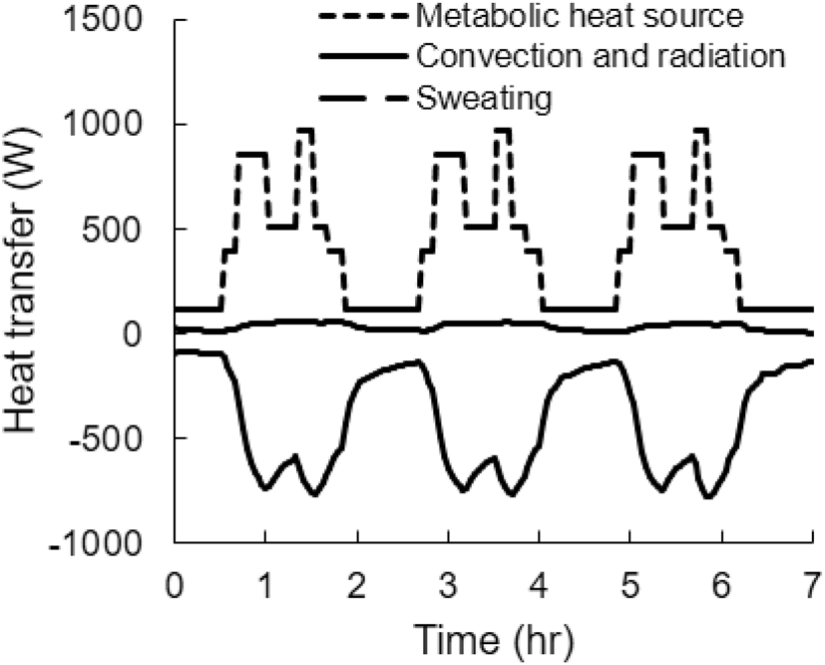
Heat exchange via the surface of the body through convection, radiation, and sweating, as well as the net heat generated in the body due to metabolic activity, for *Study* 2a with T-shirt/shorts at 36 °C and 30% humidity

Figure 10 shows the time-dependent changes in the metabolic heat source and the different modes of heat transfer during the heat-stress condition corresponding to that in Fig. 1a. The metabolic heat source, which consists of the basal metabolism and the heat generated due to exertional heat stress, closely followed the activity pattern, as the high-intensity activity was the driving force behind the metabolic heat generated in this study. Depending on the surrounding temperature, the heat generated by metabolism can be released to the environment by either sweating and/or convection and radiation. In this study condition, as well as the others, sweating dominated convection and radiation. At 36 °C, convection and radiation heated the body (Fig. 10), but the magnitude of the added heating was less than 8% of the cooling provided by sweating. Moreover, the cooling power of sweating increased with activity intensity, inversely mirroring its temporal profile. This is because the increase in core temperature signals the body’s thermoregulatory mechanism to generate more sweat, which leads to more cooling.

## Discussion

In this study, we developed an anatomically detailed 3-D thermoregulatory virtual human model representative of a 50th percentile U.S. male (Fig. 2). This FE model predicts the spatiotemporal temperature distributions throughout the human body, including 25 major organs, such as the brain, heart, lungs, liver, intestines, as well as the skeletal system, as a function of environmental conditions, physical activity, and clothing. It includes a detailed description of the various heat transfer mechanisms occurring within the body and between the body and the environment through convection, radiation, respiration, and perspiration (Fig. 3). It also includes the body’s thermoregulatory mechanisms of shivering, sweating, vasoconstriction, and vasodilation as well as the body’s internal circadian-rhythm response to the day–night cycle of temperature changes. This is in contrast with previous approaches that either represented the human anatomy very simplistically using concentric cylinders (Gagge 1973; Gagge 1971; Nishi and Gagge 1971; Stolwijk 1971; Fiala et al. 1998, 1999, 2001, 2012) or used more realistic anatomical models but did not investigate the effects of exertional heat stress on the human body (Nelson et al. 2009; Bernardi et al. 2003), which is known to be the major contributing factor to heat injuries in young, healthy adults (Stolwijk 1971; Sawka et al. 1993; Nielsen and Nielsen 1962).

We validated the 3-D thermoregulatory virtual human model, first by comparing its temperature predictions in organs and muscles under normal resting conditions and then in the rectum under strenuous heat-stress conditions. It should be noted that rectal temperature is often used as a surrogate for core body temperature under heat-stress conditions (Casa et al. 2007, 2015). Under resting conditions, we observed good agreement between model predictions and experimentally measured values for key organs, such as the brain, liver, stomach, and esophagus, with errors of less than 0.2 °C (Fig. 4). For the case of the liver and the stomach, not all subjects were healthy, as the data represented a heterogeneous mix of subjects from a biopsy diagnostic by Graf (Graf 1959). Due to the lack of other experimental data for these organs, we used the data because Graf reported that most of the subjects had normal or near-normal results, justifying our choice. In spite of such a heterogeneous population, our predicted mean liver and stomach temperatures were within 0.2 °C of the reported mean values. Although the temperature of the liver in this study was measured at only one point for each subject, Graf reported that the temperature measurements at different points within the same liver varied by less than 0.1 °C, indicating a fairly uniform temperature distribution inside the liver under normal resting conditions. We observed a similar response in the predicted spatial temperature distribution within the liver of the virtual human, where ~ 95% of the liver was within 0.1 °C, serving as an additional validation for the temperature distribution within the liver. For the case of the bladder, the prediction error was relatively high (0.4 °C); however, the predicted temperature values were well within the 95% confidence interval of the measured mean value.

It should be noted that very few studies have reported experimental measurements of organ-specific temperature (Fig. 4), owing to the difficulty in accurately measuring organ temperature using existing techniques. Our computational model seeks to address this issue by not only predicting organ temperature during normal resting conditions (for which we observed good agreement between model predictions and experiments) but also under heat-stress conditions involving intense activity, which is not practical to measure experimentally. Although, a few anatomically realistic computational models for human thermoregulation do exist in the literature (Nelson et al. 2009; Bernardi et al. 2003; Moore et al. 2014), none of the previous works have been validated with respect to key organ temperatures (e.g., brain and liver), which is one of the novelties of this work. The need for such an anatomically realistic computational model capable of accurately predicting the temperature distribution throughout the human body can help to fill this gap. In fact, our computational model complements experimental studies, helping characterize the rise in organ temperature due to environmental and exertional conditions and, in the future, helping identify novel cooling strategies to mitigate the adverse effects of heat-related illnesses.

Apart from organ-temperature validation, we also compared the predicted arm- and leg-muscle temperature response at various depths with experimental data under normal resting conditions. As would be expected, the temperature was high near the bone, and gradually dropped as we moved outwards to the skin, where body heat is lost to the atmosphere via external convection, radiation, and evaporation. We observed a good match for the temperature variation within the vastus medialis muscle (Fig. 5a, RMSE = 0.2 °C). For the triceps brachii, the temperature predictions at 10 and 25 mm from the humerus bone were within the 95% confidence interval of the measured mean values, except for a discrepancy observed at the 40-mm location (Fig. 5b, RMSE = 0.5 °C). Such a discrepancy could be due to localized differences in fat-layer thickness in the experimental subjects when compared to the virtual human, as well as the variability in the sensor location below the skin surface. A thicker fat layer is expected to provide more insulation and would result in a higher temperature. As for the sensor location, we tried to accurately match the position of the sensor in the virtual human with the one described in the experiment, however, there may be a mismatch. Because of the high spatial dependency of muscle temperature near the skin surface, any discrepancy in the location of the sensor is likely to result in distinct temperature values. Nonetheless, we observed good agreement between model predictions and experimental values at other muscle sites in the leg and the arm.

For comparing the predicted values in the rectum with experimental measurements, we used three separate studies, including a range of time-varying exertional activity levels, different environmental humidity, temperature, and wind-speed values, and different clothing. For each of the three studies, we observed a very good agreement between the model predictions and the experimental measurements (the RMSE values ranged from 0.1 to 0.3 °C, Figs. 1 and 6), indicating that our model can accurately infer core body temperature across a range of conditions. In addition to using existing literature data to validate our models, as in *Study* 2b (walking on a treadmill, Fig. 6a) (Kazman et al. 2015) and *Study* 2c (pedaling on a bicycle ergometer, Fig. 6b) (Stolwijk 1971), we performed a new study (*Study* 2a, strenuous physical activity on a treadmill) to ensure that we tested our model in scenarios that led to a substantial and nonmonotonic increase in core body temperature for a prolonged period of time (Fig. 1). In *Study* 2a, subjects performed three 80-min exercise bouts with a rest period of 50 min between each bout, under four different conditions: two environmental (30 °C and 60% RH, 36 °C and 30% RH) and two clothing (T-shirt/shorts and ACU). For each condition, we observed considerable variation in temperature distribution with time throughout the body, which is the result of internal heat generation from metabolic activity, transfer of heat within the body, and transfer of heat between the body and the environment. During physical activity, exertional heat is mostly generated in the muscles of the torso and the upper leg, which is then transported to the surface of the body through conduction and convection (i.e., through blood perfusion). This high metabolic activity in the torso along with the torso’s low surface-to-volume ratio caused a rise in its temperature (Fig. 8). In contrast, the temperature in the peripheral regions was cooler, because of their low-heat generation capacity as well as their large surface-to-volume ratio, which enabled greater heat loss to the environment.

For each of the four conditions in *Study* 2a, as expected, the predicted and measured rectal temperature increased during the exercise bouts and decreased during the rest periods between bouts (Fig. 1). While the predicted rectal temperature increased slightly with each successive bout and rose by as much as 0.3 °C between bouts, both the predicted and measured rectal temperature values were not affected by changes in environmental or clothing conditions. We believe that the presence of high-speed wind (2.5 m/s) generated by the fan diminished the barrier to sweat evaporation from the clothing’s evaporative resistance, leading to similar rise in core-body temperature for the various conditions in *Study* 2a. However, we expect a different outcome in the absence of forced winds, with the clothing exhibiting a stronger influence on the core-body temperature for the same study conditions.

To tease out the contribution of physical activity in the increase of core body temperature, we carried out a simulation for the conditions corresponding to those in Fig. 1a but without any physical activity (i.e., we set MET = 1 as an input to the model). After 6 h, starting from a pre-activity state, the maximum increase in core body temperature was 0.7 °C, instead of the 2.4 °C increase predicted under the combined exertional and environmental heat-stress conditions in Fig. 1a. After discounting for the 6-h effect of circadian rhythm on the predictions, we found that, consistent with prior observations (Stolwijk 1971; Sawka et al. 1993; Nielsen and Nielsen 1962), physical activity alone contributed to 94% of the temperature rise in the rectum, with the remaining 6% coming from the elevated temperature and moderately high relative humidity.

Our model can also be used to answer “what-if” research questions that cannot be addressed through experiments alone. For example, what are the specific contributions of the different heat-transfer mechanisms on the body’s thermal response to the conditions in Fig. 1a discussed above? Based on our simulations (Fig. 10), we determined that the metabolic heat generated by the body ranged from 115 to 970 W. In contrast, sweating caused a heat loss of 780 W during peak activity while, during the same period, convection and radiation caused a heat gain of 50 W due to the elevated environmental conditions. Interestingly, sweating, which is modulated by temperature changes in the hypothalamus and the skin from their baseline values, closely followed the temporal profile of the metabolic heat source and was ten times larger than the combined heat gain by convection and radiation. The relatively minor contributions of the convection and radiation heat-transfer mechanisms to the total heat balance within the body explain why the core temperature was largely independent of the environmental heat stress.

Rectal-temperature measurements are considered to be accurate and less prone to measurement errors than alternative means of estimating core body temperature (Casa et al. 2007; Moran and Mendal 2002). However, it is also known that there is considerable temperature variation in the rectal cavity (Miller et al. 2017; Lee et al. 2010; Buono et al. 2014). For instance, Lee et al. reported that the rectal temperature at a 4-cm depth from the anal sphincter is lower than the temperature at a 13-cm depth by at least 0.3 °C (Lee et al. 2010). Similarly, Buono et al. showed that the rectal temperature measured at a depth of 4 cm from the anal sphincter is lower than the temperature measured at depths of 7, 13, and 15 cm (Buono et al. 2014). In agreement with these studies, our model predictions showed that rectal-cavity temperature varied with cavity depth (Fig. 7a). They also showed that, at depths of 6 and 13 cm, the temperature response time was faster than that at depths of 8 and 10 cm. Moreover, in addition to the depth, we observed that temperature in the rectum also depends on the distance from the wall of the cavity. The peak rectal temperature at the wall was higher than the peak temperature at any location within the rectal wall by as much as 0.3 °C and increased at a faster rate than the temperature inside the lumen (Fig. 7b). This behavior is due to the presence of metabolic activity and perfusion of blood in the rectal wall, in contrast to the lumen. Furthermore, as heat conduction is the only mode of heat transfer within the lumen, the temperature differences decreased and eventually became negligible as we moved away from the wall. These results, which show that the temperature response can vary by as much as 0.5 °C even for adjacent regions of the body, further highlight the importance of accurately representing the human anatomy in thermoregulatory models.

In addition to the core body temperature, our 3-D FE model provides spatiotemporal temperature distribution information in the major organs of the human body, which cannot be experimentally measured or attained by thermoregulatory models with a simplistic anatomical representation (Gagge 1973; Gagge 1971; Nishi and Gagge 1971; Stolwijk 1971; Fiala et al. 1998, 1999, 2001, 2012). This gives us the unique ability to assess the thermal load at the organ level, and at the different parts of an organ, for any heat-stress condition. For example, in the simulations corresponding to Fig. 1a in *Study* 2a, the temperature at the muscular regions of the heart was higher than the temperature at the heart cavity, which was assumed to be filled with blood in our model. In addition, the temperature of the muscles at the left ventricle were as much as 0.2 °C higher than the temperature at the right ventricle during physical activity, because the thicker left ventricular wall leads to greater net heat generation when compared to the right ventricle. We also observed that the maximum volume-averaged temperature in the major organs was consistently higher than the predicted maximum rectum temperature (Table 4), in agreement with previous studies (Jardine 2007; Cheshire 2016). Moreover, the peak temperature in each organ was higher than the predicted peak temperature in the rectum. For example, the maximum temperature in the heart and the liver exceeded that of the rectum by 0.6 °C, and at 5.8 h into the heat-stress challenge, when these organs reached their peak-temperature values (Fig. 9), 100% of the volumes of the heart and the liver were above 38.5 °C, the lower-limit temperature for the onset of heat injury (Laxminarayan et al. 2018).

To demonstrate the benefit of the increased spatial resolution of the 3-D virtual human model, we repeated the simulations for *Study* 2a and predicted the core-body temperature using a cylinder model (Fiala et al. 1998). We constructed the cylinder model geometry and implemented the model equations using the commercial FE software COMSOL v5.4. To this end, we compared and contrasted the results from the cylinder model with the measured data and the 3-D model predictions. Figure S1 in the online Supplementary Material shows the core-body temperature predictions from the cylinder model and the 3-D model, along with the experimental measurements. First, we observed that our 3-D model consistently provided lower RMSEs (0.2–0.3 °C) against the experimental data when compared to the results from the cylinder model, which yielded errors two- to threefold larger (0.5–0.6 °C). Second, when we compared the maximum temperature difference (ΔT_max_) between the experimental data and model predictions (Table S2, in the online Supplementary Material), we consistently observed that the Δ*T*_max_ for the cylinder model was as much as two to three times higher than its 3-D counterpart (0.4 vs. 1.1 °C), indicating that the worst 3-D model predictions were considerably closer to the measured data. These differences in prediction accuracy are substantial (16 vs. 44%), when we consider that the range of the changes in the measured core-body temperature during the entire study was ~ 2.5 °C. Third, when we qualitatively compared the trends in core temperature predictions between the two models, the 3-D model more closely followed the experimental trends. Finally, when we performed an in-between comparison of the simulation results from the two models, we observed a sizeable difference between the two predictions (RMSE: 0.6 °C, Δ*T*_max_: 1.2 °C). The observed improvement in the 3-D model over the cylinder model arises mainly from the differences in the geometrical details, the larger heat capacity in our model (257 vs. 237 kJ/K), and the associated material properties considered in the two models.

Our study has limitations. First, we did not explicitly represent the vasculature of the human body in our model and did not consider the spatial variation in the blood temperature as well as the countercurrent heat exchange between arteries and veins. Instead, we considered heat transfer between the tissue and the blood via the Pennes bioheat transfer equation. While such an assumption might not influence the blood temperature within the torso, it might affect the temperature of the blood in the peripheral regions of the body, in particular during rapid cooling. However, the contribution from the countercurrent heat exchange is expected to be minimal during a heat-stress condition (Nelson et al. 2009; Brinck and Werner 1994). The results from our simulation show that the impact of these assumptions on the core body temperature during heat stress is negligible, as demonstrated by the good agreement with experimental data in each of the three validation studies. Second, we validated our predictions of organ temperature only for normal resting conditions and not for heat-stress conditions, owing to the unavailability of such experimental data in the literature as measuring organ-specific temperature in vivo under strenuous human activity is challenging. Third, our model did not consider acclimatization effects of the human body and the associated changes in the thermoregulatory responses. Finally, our model is based on a 50th percentile U.S. male and, therefore, does not account for variations in body size or body-fat percentage. In spite of these limitations, the model results are still valid and can help us better understand the potential risk of heat injury to humans due to exertional and environmental heat stressors.

## Conclusion

We developed an anatomically accurate 3-D thermoregulatory computational model to predict the spatiotemporal distribution of temperatures in the human body during exertional and environmental heat-stress conditions. We validated the model by comparing experimentally measured temperature with the predicted temperature in the major organs (brain, liver, stomach, bladder, and esophagus), within intramuscular regions, and in the rectum. While we performed the organ- and muscle-temperature validation using experimental measurements under normal resting conditions, we performed validation of rectal temperature using experimental measurements from three diverse heat-stress conditions, covering a range of physical activities, environmental conditions, and clothing. We showed that for the heat-stress conditions, the predicted peak temperature in key organs, such as the heart, brain, liver, and kidney, exceed the peak temperature in the core, and that in large fractions of these organs their temperature is higher than that of the core for prolonged periods of time. Hence, this new capability can help stratify the risk of organ injury for any combination of exertional and environmental heat-stress conditions. We envision that this new modeling framework can be extended, by incorporating countercurrent heat exchange between arteries and veins, an improved form of the bioheat equation, and modified thermoregulation distributions and convective heat transfer coefficients, to compare and contrast the efficacy of different whole-body or localized cooling strategies in reducing the heat load to major organs and maintaining organ integrity.

## Supplementary Information

Below is the link to the electronic supplementary material.

Supplementary file1 (PDF 743 kb)

## Data Availability

Data presented in this manuscript and the 3-D virtual human FE code will be made available through a written request (including a summary of the planned research) to the corresponding author.

## Abbreviations

*A*_sk_: Skin surface area (m^2^)
act: Amount of activity performed (MET)
${\text{act}}_{{{\text{bas}}}}$: Basal activity (MET)
$a_{{{\text{cs}}}}$: Distribution coefficient for constriction (dimensionless)
$a_{{{\text{dl}}}}$: Distribution coefficient for dilation (dimensionless)
$a_{{{\text{sw}}}}$: Distribution coefficient for sweating (dimensionless)
$a_{m,w}$: Distribution coefficient for exertional heat (dimensionless)
$a_{{{\text{sh}}}}$: Distribution coefficient for shivering (dimensionless)
$c_{p}$: Specific heat capacity (J.kg^−^^1^.K^−^^1^)
$c_{p,b}$: Specific heat capacity of blood (J.kg^−^^1^.K^−^^1^)
$C_{{{\text{met}}}}$: Circadian function for metabolic heat generation (dimensionless)
$C_{{{\text{sbf}}}}$: Circadian function for skin blood flow (dimensionless)
$C_{{{\text{rsp}}}}$: Dry heat loss due to respiration (W)
CS: Effector of constriction (dimensionless)
${\text{DL}}$: Effector of dilation (W.K^−^^1^)
*E*_rsp_: Latent heat exchange due to respiration (W)
$f_{{{\text{cl}}}}^{*}$: Local clothing area factor (dimensionless)
*H*: Exertional heat stress (W)
$h_{c}$: Convective heat-transfer coefficient (W.m^−^^2^.K^−^^1^)
$h_{r}$: Radiative heat-transfer coefficient (W.m^−^^2^.K^−^^1^)
*i*: Index for body part (dimensionless)
$I_{\text{cl}}^{*}$: Local thermal resistance of clothing layer (W^−^^1^.m^2^.K)
$i_{\text{cl}}^{*}$: Local, garment-oriented, moisture permeability index (dimensionless)
$$k$$: Thermal conductivity (W.m^−^^1^.K^−^^1^)
$L_{a}$: Lewis constant for air (0.0165 K.Pa^−^^1^)
$M_{{\text{bas,0}}}$: Basal metabolism in human body (W)
$m_{{{\text{sw}}}}$: Sweat mass (kg)
$n$: Direction normal to skin surface (dimensionless)
$p_{{{\text{amb}}}}$: Partial vapor pressure of surrounding air (Pa)
$p_{{{\text{sk}}}}$: Water vapor pressure at skin surface (Pa)
$p_{{\text{osk,sat}}}$: Saturated vapor pressure inside skin surface (Pa)
$Q_{m}$: Metabolic heat generation (W.m^−^^3^)
$Q_{m,0}$: Average basal metabolic heat generation (W.m^−^^3^)
$Q_{{{\text{shivering}}}}$: Heat generated in muscle due to shivering (W)
${{R}}_{{{E,\text{sk}}}}$: Skin moisture resistance (W^−^^1^.m^2^.Pa)
${\text{SH}}$: Heat generation due to shivering (W)
${\text{SW}}$: Rate of sweating (kg.s^−^^1^)
$T$: Body temperature dependent on spatial location and time (°C)
$$T_{0}$$: Baseline temperature (°C)
$$T_{{{\text{amb}}}}$$: Ambient temperature (°C)
$T_{b}$: Arterial blood temperature (°C)
$$T_{{{\text{hy}}}}$$: Hypothalamus temperature (°C)
$T_{{{\text{osk}}}}$: Temperature within skin surface (°C)
$T_{{{\text{sk}}}}$: Skin surface temperature (°C)
$T_{{{\text{sk}},0}}$: Baseline skin temperature (°C)
$T_{{{\text{sk}},m}}$: Area-averaged skin temperature (°C)
*T*_sr,__*m*_: Mean radiant temperature (°C)
$$t$$: Time (s)
$U_{E,\text{cl}}^{*}$: Evaporative heat-transfer coefficient (W.m^−^^2^.Pa^−^^1^)
${\text{V}}$: Volume of body tissue (m^3^)
$$v_{a}$$: Air velocity (m.s^−^^1^)
$$V_{m}$$: Muscle volume (m^3^)
VO_2_: Rate of oxygen consumption per unit mass of body (ml.min^−^^1^.kg^−^^1^)
$\Delta T_{{{\text{hy}}}}$: Difference between current and baseline hypothalamus temperatures (°C)
$\Delta T_{{{\text{sk}},m}}$: Difference between current and baseline mean skin temperatures (°C)
${\sigma }$: Stefan–Boltzmann constant (W.m^−^^2^.K^−^^4^)
$${\varepsilon }$$: Emissivity (dimensionless)
${\eta }$: Conversion efficiency of energy generated by human body to mechanical work (dimensionless)
$\lambda_{{{\text{H}}_{{2}} {\text{O}}}}$: Heat of vaporization of water (kJ.kg^−^^1^)
$$\rho$$: Density (kg/m^3^)
$\rho_{b}$: Blood density (kg/m^3^)
${\psi }$: View factor (dimensionless)
$\omega_{b}$: Blood perfusion rate (m^3^.s^−^^1^.m^−^^3^)
$\omega_{{b,{\text{bas}}}}$: Basal blood perfusion rate (﻿m^3^.s^−^^1^.m^−^^3^)
