# Supplementary material for: A 3-D virtual human thermoregulatory model to predict whole-body and organ-specific heat-stress responses
Source: Eur J Appl Physiol. 2021 Jun 5;121(9):2543–62. doi: 10.1007/s00421-021-04698-1 (PMC8357720; doi:10.1007/s00421-021-04698-1)
Supplement: Supplementary file 1 — Supplementary file1 (PDF 743 kb) [file 421_2021_4698_MOESM1_ESM.pdf]

A 3-D virtual human thermoregulatory model to predict whole-body and organ-specific heat-stress responses

European Journal of Applied Physiology

Ginu Unnikrishnan[1,2†], Rajeev Hatwar[1,2†], Samantha Hornby[1,2], Srinivas Laxminarayan[1,2], Tushar Gulati[1,2], Luke N. Belval[3], Gabrielle E.W. Giersch[3], Josh B. Kazman[4], Douglas J. Casa[3], and Jaques Reifman[1*]

[1]Department of Defense Biotechnology High Performance Computing Software Applications Institute, Telemedicine and Advanced Technology Research Center, United States Army Medical Research and Development Command, Fort Detrick, MD 21702

[2]The Henry M. Jackson Foundation for the Advancement of Military Medicine, Inc., 6720A Rockledge Drive, Bethesda, MD 20817

[3]Korey Stringer Institute, University of Connecticut, Storrs, 2095 Hillside Road U-1110, Storrs, CT 06269

[4]Consortium for Health and Military Performance, Department of Military and Emergency Medicine, F. Edward Hébert School of Medicine, Uniformed Services University, Bethesda, MD 20814

[†]Equal contribution

*Correspondence:
Jaques Reifman
Department of Defense Biotechnology High Performance Computing Software Applications Institute
Telemedicine and Advanced Technology Research Center
U.S. Army Medical Research and Development Command
FCMR-TT
504 Scott Street
Fort Detrick, MD 21702-5012
E-mail: jaques.reifman.civ@mail.mil
Phone: + 1 301 619 7915
Fax: +1 301 619 1983

THERMOREGULATION

Vasomotion. We represented the effectors of vasodilation DL (Eq. S1) and vasoconstriction CS (Eq. S2) in the virtual human model as a function of the difference between the current and the baseline temperatures of the skin $\Delta T_{sk,m}$ and the hypothalamus ΔT_{hy} [1, 2], as follows

$$DL = 21\left[\tanh\left(0.79\Delta T_{sk,m} - 0.70\right) + 1\right]\Delta T_{sk,m} + 32\left[\tanh\left(3.29\Delta T_{hy} - 1.46\right) + 1\right]\Delta T_{hy} \quad \text{(S1)}$$

$$CS = 35\left[\tanh\left(0.29\Delta T_{sk,m} + 1.11\right) - 1\right]\Delta T_{sk,m} - 7.7\Delta T_{hy} + 3.0\Delta T_{sk,m}\frac{\mathrm{d}T_{sk,m}}{\mathrm{d}t}^{(-)} \quad \text{(S2)}$$

Vasodilation and vasoconstriction influence the amount of blood flowing to the skin tissues and, thereby, the perfusion rate of blood to the skin $\omega_{b,skin}$, as given below

$$\omega_{b,i,skin} = \frac{\omega_{b,bas,skin} + [a_{dl,i} \,/\, (\rho_b c_{p,b})]DL}{1 + a_{cs,i}CS.\mathrm{e}^{-\frac{DL}{50}}}\, 2^{\left(\frac{T_{sk,i} - T_{sk,i,0}}{10}\right)} \quad \text{(S3)}$$

where the subscript 'i' denotes a part of the body, subscript 'bas' represents the basal condition, $a_{dl,i}$ refers to the coefficient for dilation [2], and $a_{cs,i}$ indicates the coefficient for constriction [2].

Sweating. We captured the thermoregulatory effectors of sweating SW (Eq. S4) as follows [1,2]

$$SW = [0.8\tanh(0.59\Delta T_{sk,m} - 0.19) + 1.2]\Delta T_{sk,m} + [5.7\ \tanh(1.98\Delta T_{hy} - 1.03) + 6.3]\Delta T_{hy} \quad \text{(S4)}$$

Sweating leads to a loss of heat from the skin surface through evaporation. The total sweat produced in the body is distributed across the body's surface, as shown by the following formula [2]

$$\frac{\mathrm{d}m_{sw,i}}{\mathrm{d}t} = a_{sw,i}SW\, 2^{\left(\frac{T_{sk,i} - T_{sk,i,0}}{10}\right)} \quad \text{(S5)}$$

where $m_{sw,i}$ denotes the mass of sweat produced in the ith body part, $a_{sw,i}$ indicates the coefficient of sweat in the ith body part [2], SW is the total rate of sweating, $T_{sk,i}$ represents the skin temperature in the ith body part, and $T_{sk,i,0}$ refers to the baseline skin temperature. The total heat transfer due to evaporation from the skin is given by the following expression [2]

$$U^*_{E,cl}(p_{sk} - p_{amb}) = \frac{\lambda_{H_2O}}{A_{sk}}\frac{dm_{sw}}{dt} + \frac{p_{osk,sat} - p_{sk}}{R_{E,sk}} \tag{S6}$$

where $U^*_{E,cl}$ denotes the evaporative coefficient, p_{sk} indicates the water vapor pressure at the skin surface, p_{amb} represents the vapor pressure of the surrounding air, λ_{H_2O} = 2,260 kJ kg^{-1} refers to the heat of vaporization of water, A_{sk} indicates the skin surface area, $p_{osk,sat}$ denotes the saturated vapor pressure inside the skin surface, and $1/R_{E,sk}$ = 0.003 W.m^{-2}.Pa^{-1} represents the moisture permeability of the skin. The saturated vapor pressure inside the skin surface is given by the following expression [2]

$$p_{osk,sat} = 100 \exp\left[18.965 - \frac{4030}{T_{osk} + 235}\right] \tag{S7}$$

where T_{osk} denotes the temperature within the skin surface. The evaporative coefficient $U^*_{E,cl}$, which takes into consideration the effect of clothing, is given as

$$U^*_{E,cl} = \frac{L_a}{\sum_{j=1}^{J}\left(\frac{I^*_{cl}}{i^*_{cl}}\right)_j + \frac{1}{f^*_{cl} \cdot h_c}} \tag{S8}$$

where I^*_{cl} represents the local heat resistance of the jth clothing, i^*_{cl} represents the local, garment-oriented, moisture permeability index, L_a refers to the Lewis constant for air, f^*_{cl} denotes the

local clothing area factor, and h_c represents the convective heat-transfer coefficient. For more details on the clothing parameters, please refer to Table 3.

The value of p_{sk}, which can be obtained by rearranging Eq. (S6), is given as

$$p_{sk} = \frac{\frac{\lambda_{H_2O}}{A_{sk}} \frac{dm_{sw}}{dt} + \frac{p_{osk,sat}}{R_{E,sk}} + p_{amb} U_{E,cl}^{*}}{U_{E,cl}^{*} + \frac{1}{R_{E,sk}}} \tag{S9}$$

In this study, we applied Eq. (S3) to the skin surface as a boundary condition to simulate the net heat exchange at the surface due to evaporation.

Shivering. The effectors of shivering *SH* (Eq. S10) were represented as a function of the difference between the current and the baseline temperatures of the skin $\Delta T_{sk,m}$ and the hypothalamus ΔT_{hy} [1, 2], as follows

$$SH = 10\left[\tanh\left(0.48\Delta T_{sk,m} + 3.62\right) - 1\right]\Delta T_{sk,m} - 27.9\Delta T_{hy} - 28.6 + 1.7\Delta T_{sk,m}\frac{dT_{sk,m}}{dt}^{(-)} \tag{S10}$$

where $dT_{sk,m}/dt$ denotes the rate of change of the average skin temperature and the negative sign indicates that the rate of change is activated only in cold environmental conditions. Shivering causes heat to be generated in the system during cold stress. The shivering heat *SH* is distributed throughout the entire body based on the distribution coefficients a_{sh} [2], as described below

$$Q_{shivering,i} = a_{sh,i}\frac{SH}{V_{m,i}} \tag{S11}$$

where $Q_{shivering,i}$ denotes the heat generated by the muscle in the ith body part and $V_{m,i}$ represents the muscle volume of that part.

Table S1: Thermophysical properties of different tissue types used in the model (from [2, 3]).

Tissue	ρ (kg.m^{-3})	k (W.m^{-1}.K^{-1})	c_p (J.Kg^{-1}.K^{-1})	ω_{b0} (l.s^{-1}.m^{-3})	Q_{m0} (W.m^{-3})
Adrenal gland	1,028	0.44	3,513	25.00	23,197
Bladder	1,086	0.52	3,581	1.41	1,312
Brainstem	1,046	0.51	3,630	9.73	11,884
Cerebrum	1,046	0.51	3,630	9.73	11,884
Cerebellum	1,045	0.51	3,653	13.40	16,373
Cerebrospinal fluid	1,007	0.57	4,095	0.00	0
Esophagus lumen	1	0.03	3,500	3.29	3,060
Esophagus wall	1,040	0.53	3,500	3.29	3,060
Eyes	1,005	0.59	4,047	0.00	0
Gallbladder	1,071	0.52	3,716	0.53	497
Gallbladder lumen	928	0.58	4,037	0.00	0
Heart	1,081	0.56	3,686	18.50	42,640
Heart lumen	1,050	0.52	3,617	0.00	0
Hypothalamus	1,045	0.55	3,696	13.30	16,231
Kidney	1,066	0.53	3,763	67.40	19,248
Large intestine lumen	1,045	0.56	3,801	0.00	0
Large intestine wall	1,088	0.54	3,654	13.90	12,894
Liver	1,055	0.51	3,600	15.50	10,713
Lungs	1,050	0.14	3,600	2.63	816
Muscle	1,040	0.56	3,600	0.34	502
Pancreas	1,087	0.51	3,164	13.90	12,914
Prostate	1,045	0.51	3,760	6.86	6,378
Skeleton-cartilage	1,100	0.49	3,568	0.64	298
Skeleton-flat	1,397	0.31	1,986	0.56	256
Skeleton-long	1,537	0.27	1,614	0.46	210
Skeleton-mandible	1,397	0.31	1,986	0.56	256
Skeleton-short	1,178	0.31	2,274	0.59	271
Skeleton-skull	1,397	0.31	1,986	0.56	256
Skeleton-vertebrae	1,178	0.31	2,274	0.59	271
Skin-dermis	1,109	0.52	3,390	1.44	1827
Skin-epidermis	1,109	0.26	3,600	0.00	0
Small intestine lumen	1,045	0.56	3,801	0.00	0
Small intestine wall	1,030	0.49	3,595	17.60	8,185
Spinal cord	1,075	0.51	3,630	2.87	2,669
Spleen	1,089	0.53	3,630	28.30	15,755
Stomach	1,088	0.53	3,690	8.35	7,756
Stomach lumen	1,045	0.56	3,801	0.00	0
Subarachnoid space	1,007	0.57	4,096	0.00	0
Subcutaneous fat[‡]	911	0.26	1,920	0.33	282
Testis	1,082	0.52	3,778	3.60	3,348
Thymus	1,023	0.34	3,043	4.21	3,915

Trachea	1,080	0.49	3,568	0.63	585
Trachea lumen	1	0.03	1,004	0.00	0
Vertebral disc	1,100	0.49	3,568	0.64	596

‡ The thermophysical properties of fat were adjusted to take into account the addition of a skin layer

COMPARISON BETWEEN VIRTUAL HUMAN AND CYLINDER MODEL

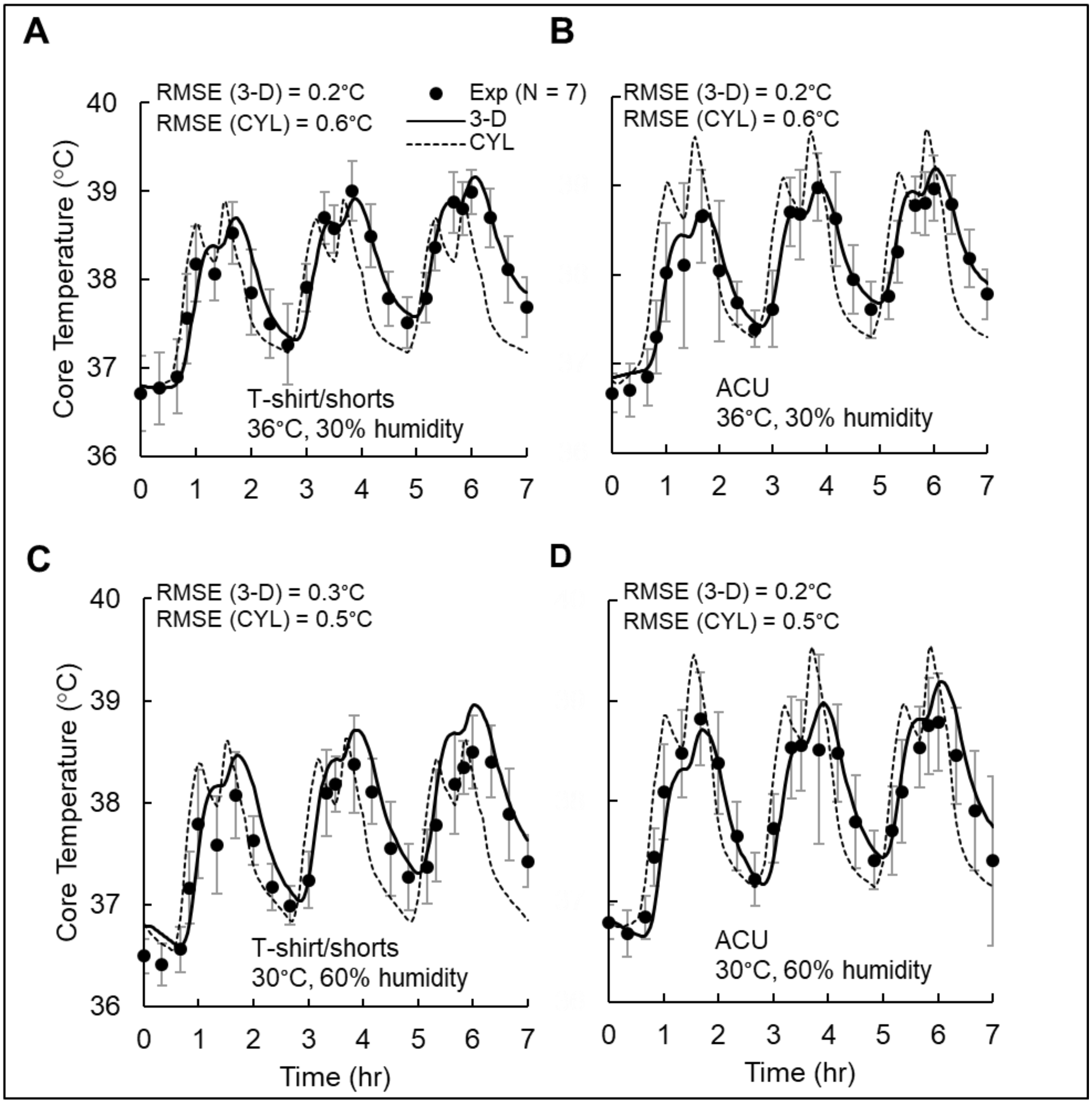

Fig. S1: Virtual human (3-D) vs. cylinder (CYL) model comparison for *Study* 2a in which seven subjects perform three extraneous, time-varying exercise bouts under four different conditions. A rectal probe served to measure core temperature. The clothing and environmental conditions varied across the four exercise conditions: (A) T-shirt/shorts at 36°C and 30% humidity, (B)

active combat uniform (ACU) at 36°C and 30% humidity, (C) T-shirt/shorts at 30°C and 60% humidity, and (D) ACU at 30°C and 60% humidity. Filled circles denote the mean experimental values (N = 7), while the vertical bars represent one standard error of the mean. Solid line denotes the 3-D model predictions, while the dashed line represents cylinder model predictions. RMSE: root mean squared error.

Table S2: Errors in core-body temperature predictions from the virtual human (3-D) and cylinder (CYL) model

	Model	(A)[c] Very Hot and Dry, T-shirt/shorts	(B)[c] Very Hot and Dry, ACU	(C)[c] Hot and Humid, T-shirt/shorts	(D)[c] Hot and Humid, ACU
			Model vs Experiment		
RMSE (°C)	3-D	0.2	0.2	0.3	0.2
	CYL	0.6	0.6	0.5	0.5
ΔT_{max} (°C)	3-D	0.5	0.4	0.6	0.5
	CYL	1.3	1.1	1.0	0.9
			3-D vs CYL		
RMSE[a] (°C)		0.6	0.6	0.6	0.6
ΔT_{max}[b] (°C)		1.3	1.2	1.2	1.2

[a]Root mean squared error
[b]Maximum temperature difference between model prediction and measured data
[c]Designations A, B, C, and D refer to Figure S1

REFERENCES

1. Fiala, D., G. Havenith, P. Bröde, B. Kampmann, and G. Jendritzky, *Utci-Fiala Multi-Node Model of Human Heat Transfer and Temperature Regulation.* International Journal of Biometeorology, 2012. **56**(3): p. 429-41.

2. Fiala, D., *Dynamic Simulation of Human Heat Transfer and Thermal Comfort*, in *Institute of Energy and Sustainable Development* 1998, Doctoral dissertation, De Montfort University Leicester, UK.

3. Hasgall, P.A., F. Di Gennaro, C. Baumgartner, E. Neufeld, B. Lloyd, M.C. Gosselin, D. Payne, A. Klingenböck, and N. Kuster, *It'is Database for Thermal and Electromagnetic Parameters of Biological Tissues, Version 4.0*. 2018.
